# A pan-SARS-CoV-2-specific soluble angiotensin-converting enzyme 2-albumin fusion engineered for enhanced plasma half-life and needle-free mucosal delivery

**DOI:** 10.1093/pnasnexus/pgad403

**Published:** 2023-11-28

**Authors:** Sopisa Benjakul, Aina Karen Anthi, Anette Kolderup, Marina Vaysburd, Heidrun Elisabeth Lode, Donna Mallery, Even Fossum, Elisabeth Lea Vikse, Anna Albecka, Aleksandr Ianevski, Denis Kainov, Karine Flem Karlsen, Siri Aastedatter Sakya, Mari Nyquist-Andersen, Torleif Tollefsrud Gjølberg, Morten C Moe, Magnar Bjørås, Inger Sandlie, Leo C James, Jan Terje Andersen

**Affiliations:** Department of Pharmacology, Institute of Clinical Medicine, University of Oslo, Oslo 0372, Norway; Department of Immunology, Oslo University Hospital Rikshospitalet, Oslo 0372, Norway; Precision Immunotherapy Alliance (PRIMA), University of Oslo, Oslo 0372, Norway; Department of Pharmacology, Institute of Clinical Medicine, University of Oslo, Oslo 0372, Norway; Department of Immunology, Oslo University Hospital Rikshospitalet, Oslo 0372, Norway; Precision Immunotherapy Alliance (PRIMA), University of Oslo, Oslo 0372, Norway; Department of Pharmacology, Institute of Clinical Medicine, University of Oslo, Oslo 0372, Norway; Department of Immunology, Oslo University Hospital Rikshospitalet, Oslo 0372, Norway; Precision Immunotherapy Alliance (PRIMA), University of Oslo, Oslo 0372, Norway; Protein and Nucleic Acid Chemistry Division, Medical Research Council, Laboratory of Molecular Biology, Cambridge CB2 0QH, UK; Department of Pharmacology, Institute of Clinical Medicine, University of Oslo, Oslo 0372, Norway; Department of Immunology, Oslo University Hospital Rikshospitalet, Oslo 0372, Norway; Department of Ophthalmology, Oslo University Hospital and University of Oslo, Oslo 0450, Norway; Protein and Nucleic Acid Chemistry Division, Medical Research Council, Laboratory of Molecular Biology, Cambridge CB2 0QH, UK; Department of Virology, Norwegian Institute of Public Health, Oslo 0213, Norway; Department of Virology, Norwegian Institute of Public Health, Oslo 0213, Norway; Protein and Nucleic Acid Chemistry Division, Medical Research Council, Laboratory of Molecular Biology, Cambridge CB2 0QH, UK; Department of Clinical and Molecular Medicine, Norwegian University of Science and Technology, Trondheim 7491, Norway; Department of Clinical and Molecular Medicine, Norwegian University of Science and Technology, Trondheim 7491, Norway; Institute of Technology, University of Tartu, Tartu 50411, Estonia; Institute for Molecular Medicine Finland, University of Helsinki, Helsinki 00290, Finland; Department of Pharmacology, Institute of Clinical Medicine, University of Oslo, Oslo 0372, Norway; Department of Immunology, Oslo University Hospital Rikshospitalet, Oslo 0372, Norway; Department of Pharmacology, Institute of Clinical Medicine, University of Oslo, Oslo 0372, Norway; Department of Immunology, Oslo University Hospital Rikshospitalet, Oslo 0372, Norway; Precision Immunotherapy Alliance (PRIMA), University of Oslo, Oslo 0372, Norway; Department of Pharmacology, Institute of Clinical Medicine, University of Oslo, Oslo 0372, Norway; Department of Immunology, Oslo University Hospital Rikshospitalet, Oslo 0372, Norway; Precision Immunotherapy Alliance (PRIMA), University of Oslo, Oslo 0372, Norway; Department of Pharmacology, Institute of Clinical Medicine, University of Oslo, Oslo 0372, Norway; Department of Immunology, Oslo University Hospital Rikshospitalet, Oslo 0372, Norway; Precision Immunotherapy Alliance (PRIMA), University of Oslo, Oslo 0372, Norway; Department of Ophthalmology, Oslo University Hospital and University of Oslo, Oslo 0450, Norway; Department of Ophthalmology, Oslo University Hospital and University of Oslo, Oslo 0450, Norway; Department of Virology, Norwegian Institute of Public Health, Oslo 0213, Norway; Department of Biosciences, University of Oslo, Oslo 0371, Norway; Protein and Nucleic Acid Chemistry Division, Medical Research Council, Laboratory of Molecular Biology, Cambridge CB2 0QH, UK; Department of Pharmacology, Institute of Clinical Medicine, University of Oslo, Oslo 0372, Norway; Department of Immunology, Oslo University Hospital Rikshospitalet, Oslo 0372, Norway; Precision Immunotherapy Alliance (PRIMA), University of Oslo, Oslo 0372, Norway

**Keywords:** SARS-CoV-2, FcRn, ACE2, half-life, mucosal delivery

## Abstract

Immunocompromised patients often fail to raise protective vaccine-induced immunity against the global emergence of severe acute respiratory syndrome coronavirus 2 (SARS-CoV-2) variants. Although monoclonal antibodies have been authorized for clinical use, most have lost their ability to potently neutralize the evolving Omicron subvariants. Thus, there is an urgent need for treatment strategies that can provide protection against these and emerging SARS-CoV-2 variants to prevent the development of severe coronavirus disease 2019. Here, we report on the design and characterization of a long-acting viral entry-blocking angiotensin-converting enzyme 2 (ACE2) dimeric fusion molecule. Specifically, a soluble truncated human dimeric ACE2 variant, engineered for improved binding to the receptor-binding domain of SARS-CoV-2, was fused with human albumin tailored for favorable engagement of the neonatal fragment crystallizable receptor (FcRn), which resulted in enhanced plasma half-life and allowed for needle-free transmucosal delivery upon nasal administration in human FcRn-expressing transgenic mice. Importantly, the dimeric ACE2-fused albumin demonstrated potent neutralization of SARS-CoV-2 immune escape variants.

**Significance Statement**

Individuals with suppressed or compromised immune systems do not necessarily respond adequately to coronavirus disease 2019 vaccines. Consequently, severe acute respiratory syndrome coronavirus 2 (SARS-CoV-2) infection may persist in these individuals, resulting in prolonged viral shedding and the development of mutant immune-evasive variants. While monoclonal antibodies have been approved for therapy and prophylaxis, they have shown to lose efficacy against the Omicron lineages of SARS-CoV-2. As the virus requires binding to its host receptor, angiotensin-converting enzyme 2 (ACE2), the prophylactic and therapeutic use of recombinant truncated human ACE2 is an attractive strategy. However, such soluble ACE2 has a plasma half-life of only few hours, which severely hampers its utility. To meet these challenges, we report on an albumin-based protein fusion, in which truncated dimeric ACE2 was fused with human albumin engineered for enhanced engagement of neonatal fragment crystallizable receptor, providing an extended plasma half-life and allowing for efficient delivery across mucosal barriers upon nasal administration. Further incorporating an ACE2 engineered for higher affinity to SARS-CoV-2 was shown to potently block infection of Omicron subvariants. Thus, this tailored ACE2 fusion design should be attractive for strategies aiming for both local and systemic protection against SARS-CoV-2 infection.

## Introduction

The first pandemic severe acute respiratory syndrome coronavirus 2 (SARS-CoV-2) strain, which originated in Wuhan, China, and causes coronavirus disease 2019 (COVID-19), has resulted in a global health crisis (1, 2). The disease manifests itself in different forms and stages, ranging from asymptomatic viral carriage to life-threatening pneumonia and cytokine dysregulation (2). Hitherto, several vaccines have been approved and distributed globally (3, 4). Nevertheless, there are tens of millions of individuals worldwide who do not respond adequately to the vaccines, as they live with suppressed or compromised immune systems due to underlying diseases or immunosuppressive medical treatment for organ transplantation, cancer, and autoimmune diseases (5–8). In this population, SARS-CoV-2 infection can persist for several months, allowing for prolonged viral shedding and the emergence of mutant immune-evasive variants (9–12). Thus, there is a need for multiple treatment options with a variety of complementary approaches that are specifically tailored for long-term prophylactic utility by immunocompromised or high-risk infected patients. However, biologics are often rapidly cleared from the body, which hampers their therapeutic efficacy and necessitates frequent administration by intravenous (IV) infusion. This places a burden on both patients and the healthcare system. Hence, to increase the likelihood of clinical success, innovative molecular design should secure optimal pharmacokinetic properties for improved drug administration and dosing regimens.

Angiotensin-converting enzyme 2 (ACE2) has been identified as the principal host receptor for SARS-CoV-2, interacting with the receptor-binding domain (RBD) of the spike protein displayed on the viral surface (1, 13–15). Structurally, the transmembrane-bound, full-length human ACE2 is composed of extracellular domains, including the N-terminal peptidase domain (PD; residues 18–615) and the collectrin-like domain (CLD; residues 616–768), followed by a transmembrane part (residues 769–789) and a short C-terminal cytoplasmic tail (residues 790–805) (16). In host cells, the spike protein of SARS-CoV-2 binds to the N-terminal extracellular domain of the membrane-bound, full-length ACE2, thereby facilitating viral entry and replication (13–17). ACE2 is broadly expressed by lung alveolar epithelial cells, as well as in the blood vessels and tissues of the intestine, heart, and kidneys (18, 19). In addition, there are other receptors and compounds that have been reported to contribute to infection, for instance transmembrane serine protease 2 (TMPRSS2) (14) and GM1 ganglioside (20, 21), as well as the disputed CD147 (22, 23, 24).

Blocking SARS-CoV-2 infection can be achieved by neutralizing antibodies raised in response to spike-based vaccines, or by the design of long-acting monoclonal immunoglobulin G (IgG) antibodies that selectively target the spike protein of SARS-CoV-2 (3, 4, 25). Although some of these antibodies have been clinically authorized and shown to be protective, their efficacy has become diminished due to the emergence of new escape mutant variants, notably the Omicron lineages (26–31). In response, new monoclonal antibodies and alternative scaffolds, capable of neutralizing Omicron subvariants, have been developed but have not yet reached clinical use (32–35). However, there is a potential risk that these agents may also lose their efficacy when the virus continues to evolve, which poses a significant challenge to commercial development.

An alternative attractive approach is the use of a recombinant human ACE2 protein as a decoy receptor for SARS-CoV-2 by blocking the viral receptor-binding site (36–38). While the virus may undergo mutations and evade recognition by spike-specific antibodies, it must maintain the capability to engage its main host receptor. For this reason, biologic designs based on ACE2 are anticipated to be more resistant to viral mutation, evidenced by a range of studies (39–43). An example is the clinical-grade soluble, truncated dimeric human ACE2 (residues 1–740), known as APN01, which consists of the extracellular domains of the receptor, excluding the transmembrane part and the cytoplasmic tail (40, 44–48). Such glycosylated ACE2 can also be found endogenously in the blood or body fluids as a result of proteolytic cleavage of the transmembrane-bound, full-length ACE2 (36, 37, 49, 50). Prior to the pandemic, APN01 had been studied in healthy volunteers and patients with acute respiratory distress syndrome and shown to be safe (44, 45). Consequently, APN01 was immediately tested in a phase II clinical trial for COVID-19 treatment (NCT04335136), where it was well tolerated and showed a good safety profile in severely ill patients. A case report also revealed clinical improvement associated with a rapid decline in viral load and a reduction in inflammatory mediators (47). However, the treatment requires IV infusions of APN01 (0.4 mg/kg), administered twice daily for a week or longer (47). This is due to the fact that soluble dimeric human ACE2 has a plasma half-life of only 10 h (44), which is not optimal in an infection setting requiring both systemic and mucosal tissue presence. The limited half-life of APN01 may explain the less-than-expected clinical results observed in patients with acute respiratory distress syndrome (44, 45) and in those hospitalized with COVID-19 (NCT04335136). As such, this ACE2 format is not relevant for the prophylactic treatment of immunocompromised patients, and strategies for improving the pharmacokinetics of soluble recombinant human ACE2 are needed in order to increase its utility.

Several approaches for achieving half-life extension have been reported, including genetic fusion to the fragment crystallizable (Fc) part of IgG1 (51–56) or full-length human albumin (57), as well as albumin-binding domain (ABD) that can hijack endogenous albumin (58–60). However, needle-free administration of ACE2 directly to the mucosal sites, such as the respiratory and gastrointestinal tracts, represents an attractive convenient strategy to combat SARS-CoV-2 infection and transmission. Support for this has been demonstrated in dogs following intranasal (IN) administration of an inhaled formulation of APN01, which has led to initiation of a clinical phase I trial to evaluate its safety and tolerability (NCT05065645) (48). Another interesting approach is a truncated ACE2 format fused with ABD (ACE2-ABD), reported to provide enhanced survival and brain protection against SARS-CoV-2 infection following IN administration in mice (60). A recent report has also demonstrated that inhaled ACE2 fused with an IgG1 Fc fragment protects against disease in mice, hamsters, and nonhuman primates (43). Moreover, as high viral loads have been detected in the saliva of COVID-19 patients (61, 62), oral delivery is being explored for a clinical-grade nonglycosylated full-length ACE2 fused with cholera toxin B (CTB-ACE2), expressed in plant cells and delivered through a chewing gum (41, 63–66). CTB-ACE2 has shown efficacy in decreasing viral infection in nasopharyngeal swab and saliva samples from COVID-19 patients (41, 65). Currently, a phase I/II placebo-controlled, double-blinded clinical trial (IND 154897, NCT05433181, IRB 851459) is ongoing to evaluate viral load in saliva both before and after the use of CTB-ACE2 or placebo gum.

To further improve strategies based on recombinant ACE2, studies have reported on the engineering of ACE2 with improved affinity for the SARS-CoV-2 RBD, comparable with that of clinical-grade monoclonal antibodies (42, 52, 53, 67, 68). One attractive candidate harbors three amino acid substitutions (T27Y/L79T/N330Y; YTY) that enhance binding to RBD from a range of SARS-CoV-2 variants, leading to more efficient neutralization of live SARS-CoV-2 of nearly two orders of magnitude (52, 53). Such engineered ACE2 may be combined with formats and delivery strategies discussed above to provide more potent prophylactic and therapeutic candidates.

In this study, we report on engineering of soluble, truncated monomeric and dimeric ACE2 formats that are genetically fused with either full-length wild-type (WT) human albumin or an engineered human albumin variant with favorable engagement of the human form of the neonatal Fc receptor (FcRn), which is a key half-life regulator of albumin, as reviewed (69–71). We demonstrate that such ACE2-albumin fusions are well produced, and that albumin engineering for improved FcRn binding provides both an extended plasma half-life and the ability to be delivered across mucosal surfaces upon IN delivery in human FcRn-expressing mice. Importantly, we show that the dimeric nature of ACE2 is required for effective neutralization of SARS-CoV-2 infection, in which incorporation of YTY-engineered ACE2 into the albumin-based format provides a potent pan-SARS-CoV-2-specific soluble ACE2.

## Results

### Design of human albumin-based dimeric ACE2 fusion

**Fig. 1.**
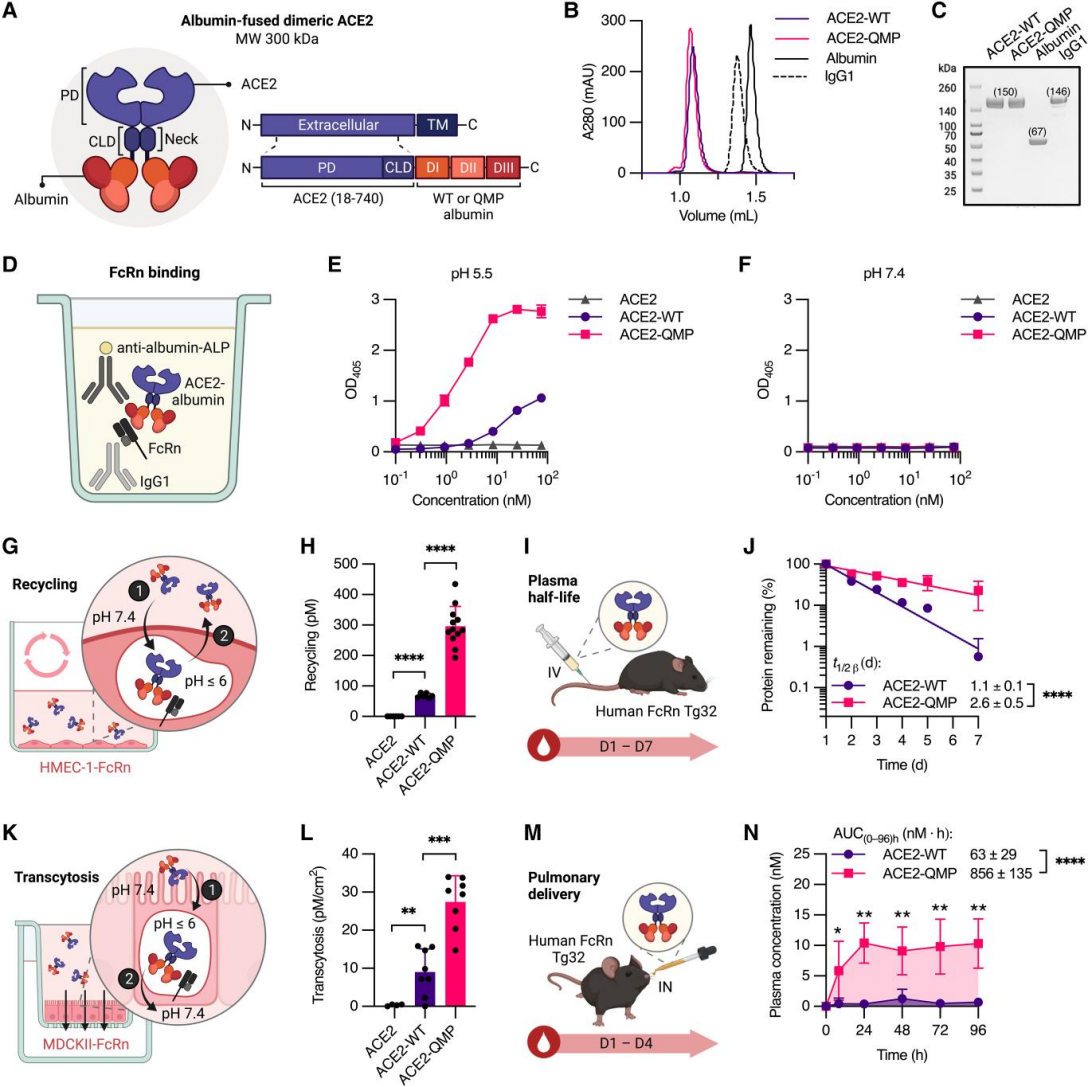
Design of human albumin-fused dimeric ACE2 with favorable pharmacokinetic and transmucosal transport properties. A) Illustration of the dimeric ACE2-albumin fusions (300 kDa), in which truncated dimeric human ACE2 (residues 18–740) is genetically fused to the N-terminal end of full-length human WT or QMP albumin. The extracellular part of ACE2 includes the PD and the neck region of CLD, where dimerization occurs. The three subdomains DI, DII, and DIII of albumin are indicated. B) Representative analytical SEC profile and C) nonreducing SDS-PAGE gel of noncovalent dimeric ACE2-WT and ACE2-QMP (300 kDa), in comparison to full-length WT human albumin (67 kDa) and human IgG1 (146 kDa). D) Illustration of the ELISA setup used to evaluate binding to human FcRn, in which a His-tagged human FcRn is captured on a coated human IgG1 mutant variant (MST/HN), followed by binding of the dimeric ACE2-albumin fusion and detection with an ALP-conjugated anti-human albumin antibody. E and F) Representative ELISA results showing the pH-dependent binding of unfused ACE2, ACE2-WT, and ACE2-QMP to human FcRn at E) pH 5.5 and F) pH 7.4 (mean ± SD, *n* = 2). G) Illustration of the HERA with adherent HMEC-1-FcRn cells used to study cellular recycling. H) The recycled amounts of ACE2-WT and ACE2-QMP, in comparison with unfused ACE2 (mean ± SD, *n* = 3; 4 independent experiments). I) Illustration of the *in vivo* experimental setup used to determine plasma half-life in homozygous human FcRn Tg32 mice by IV injection into the lateral tail vein. Isolated plasma samples from blood were collected over a period of up to 7 days. J) Representative elimination curves of ACE2-WT and ACE2-QMP post-IV administration. The data are presented as the percentage of ACE2-albumin fusions remaining in plasma compared with day 1 (mean ± SD, *n* = 5), and the average plasma half-life ( $t_{1/2\beta }$) (mean ± SEM, *n* = 5). K) Illustration of the Transwell assay with polarized epithelial cell monolayers of MDCKII-FcRn cells used to study apical-to-basolateral transcytosis. L) The transcytosed amounts of ACE2-WT and ACE2-QMP, in comparison with unfused ACE2 (gray) (mean ± SD, *n* = 4; 2 independent experiments). M) Illustration of the *in vivo* experimental setup used to evaluate pulmonary delivery in homozygous human FcRn Tg32 mice by IN administration into the nostrils. Isolated plasma samples from blood were collected over a period of up to 4 days. N) The amount of ACE2-WT and ACE2-QMP detected in plasma at 8, 24, 48, 72, and 96 h post-IN administration, and the respective AUC from a representative experiment (mean ± SD, *n* = 5). Unpaired two-tailed *t*-test was used for statistical analysis, where *P = 0.0435, **P = 0.0043, ***P = 0.0001, and ****P < 0.0001. The illustrations were created with BioRender.com.

Albumin is a soluble protein consisting of a single nonglycosylated polypeptide that lacks immune effector functions and is easy to manufacture (72). We designed recombinant ACE2-albumin fusion proteins (300 kDa), where the extracellular part of truncated dimeric human ACE2 (residues 18–740) was fused to the N-terminal end of domain I (DI) of full-length human albumin (Fig. 1A). In addition to WT albumin, we fused an engineered human albumin variant with three amino acid substitutions (E505Q/T527M/K573P; QMP) (73) to the dimeric ACE2 to form ACE2-WT and ACE2-QMP, respectively (Fig. 1A). The amino acid sequence of ACE2 is similar to that of the clinical-grade product of APN01, which excludes the transmembrane part and the cytoplasmic tail. Each ACE2 consists of a PD (residues 19–615) followed by part of CLD (residues 616–740) with a neck region (residues 616–726; Fig. 1A), the latter of which is required for dimerization (16). The recombinant fusion products were expressed well in a serum-free transient Expi293 expression system, yielding an estimated 45–160 mg/L of culture (Fig. S1). Pure, soluble fractions of noncovalent dimers, with expected molecular weight (MW), were obtained, as demonstrated by the size exclusion chromatography (SEC) profile and sodium dodecyl sulfate polyacrylamide gel electrophoresis (SDS-PAGE) gel (Fig. 1B and C). In addition to the ACE2-albumin fusions, an unfused version of the truncated dimeric ACE2 (residues 18–740) was successfully created for benchmarking using the same expression system (Fig. S1).

### Engineered QMP albumin provides ACE2 with tailored FcRn-mediated plasma half-life extension

The rationale for choosing albumin as a carrier is based on that FcRn is the key cellular regulator of albumin homeostasis (73, 74), as well as IgG, which provides both ligands with a long plasma half-life of about 3 weeks in humans (72). The receptor is widely expressed in the endothelium, such as blood vessels, where it rescues albumin from intracellular lysosomal degradation and exports the molecule back out of the cell via pH-dependent recycling process (74–76). The QMP-engineered albumin variant improves pH-dependent binding to FcRn and has been shown to provide monovalent fusion products with 2- to 4-fold longer plasma half-life than WT albumin counterparts in transgenic mice expressing human FcRn (human FcRn Tg32) (73, 77).

To verify the ability of the dimeric ACE2-albumin fusions to engage human FcRn in a pH-dependent manner, enzyme-linked immunoassay (ELISA) and surface plasmon resonance (SPR) analyses were performed, which confirmed that ACE2-QMP bound the receptor more strongly than ACE2-WT at pH 5.5 (Figs. 1D, E and S1; Table S1), while maintaining pH-dependence, with no binding detected at pH 7.4 (Figs. 1F and S1; Table S1). In contrast, unfused ACE2 did not bind FcRn under either pH condition (Fig. 1E and F). Using an *in vitro* human endothelial cellular recycling assay (HERA) with adherent human FcRn-expressing human dermal microvascular endothelial cells (HMEC-1-FcRn) (75, 78), about 4-fold more of ACE2-QMP was found to be rescued from intracellular degradation than ACE2-WT, whereas unfused ACE2 was not recycled (Fig. 1G and H).

In line with the *in vitro* data, IV injection of the fusions in human FcRn Tg32 mice resulted in a plasma half-life of 2.6 days for ACE2-QMP, which was 2.4-fold longer than that of ACE2-WT (1.1 days) (Fig. 1I and J). In the absence of albumin, soluble truncated ACE2 displayed a shorter half-life estimated to 11.5 h (Fig. S1), in line with previous reports showing half-lives of 6.8 h and 10 h in mice and humans, respectively (44, 58). As subcutaneous (SC) administration may be more convenient than IV infusion, we further explored this route of administration in the transgenic mice, which demonstrated efficient delivery of the ACE2-albumin fusions from the injection site to the blood, with ACE2-QMP possessing a plasma half-life nearly 5-fold that of ACE2-WT (Fig. S1). Importantly, the plasma concentrations of ACE2-QMP were significantly higher than that of unfused ACE2 or ACE2-WT at all time points tested following IV or SC administration (Fig. S1). Hence, the pharmacokinetic properties of soluble dimeric ACE2 could be tailored by the use of engineered human albumin with improved pH-dependent FcRn binding characteristics.

### Albumin provides FcRn-directed delivery across respiratory mucosal barriers

FcRn binds and recycles IgG as well as albumin, and the two ligands engage the receptor at distinct binding sites in a noncooperative manner (79, 80). Depending on cell type and cell-specific sorting molecule expression pattern, the ligands will be recycled or transcytosed (76). We recently revealed that albumin is more efficiently transcytosed across mucosal epithelial barriers than IgG following IN administration, and that albumin could mediate transmucosal delivery of its fusion partners, which was enhanced with the QMP albumin variant (73). Consistent with this, fusion of ACE2 with albumin resulted in transcytosis from the apical to basolateral side across a polarized epithelial monolayer of human FcRn-expressing Madin-Darby canine kidney II (MDCKII-FcRn) cells *in vitro*, whereas unfused ACE2 was not transcytosed (Fig. 1K and L). Moreover, the transepithelial transport of ACE2-QMP was at least twice as efficient as ACE2-WT (Fig. 1L). When the fusions were administered intranasally to human FcRn Tg32 mice, ACE2-QMP crossed the mucosal barriers and entered the bloodstream significantly more efficiently than ACE2-WT, resulting in a 13.6-fold increase in the area under the concentration–time curve (AUC) over 4 days (Fig. 1M and N). In line with the *in vitro* data, unfused ACE2 was not detected in plasma at any time points (Fig. S1). Thus, direct fusion of dimeric ACE2 with engineered QMP albumin variant improved both pharmacokinetic and transmucosal transport properties.

### The dimeric ACE2-albumin fusion design effectively blocks SARS-CoV-2 cellular infection

**Fig. 2.**
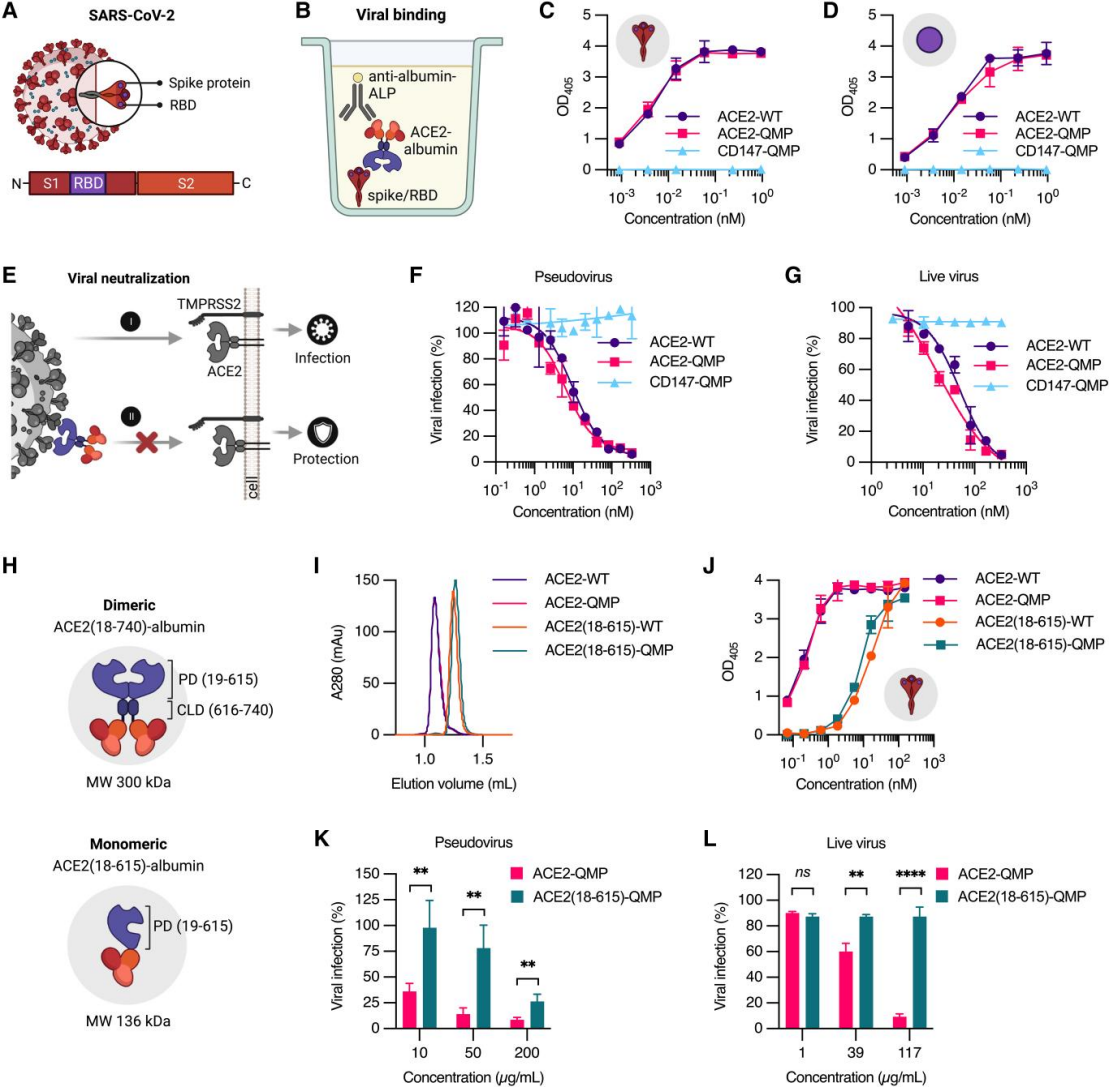
Effective binding and blockage of SARS-CoV-2 require ACE2 dimerization. A) Illustration of the SARS-CoV-2 covered by spike proteins made up of the S1 subunit with RBD and the S2 subunit. B) Illustration of the ELISA setup used to evaluate viral binding, in which a dimeric ACE2-albumin fusion is captured by the SARS-CoV-2 spike or derived RBD, and detected with an alkaline phosphayase (ALP)-conjugated anti-human albumin antibody. C and D) Representative ELISA results showing the binding of ACE2-WT and ACE2-QMP to recombinant C) spike and D) derived RBD of SARS-CoV-2 (Wuhan), in comparison with CD147-QMP (mean ± SD, *n* = 2). E) Illustration of the binding of an ACE2-albumin fusion to the spike proteins displayed on the viral surface and blocking of viral entry to cells expressing ACE2 and TMRPSS2. F and G) Representative neutralization experiments showing the capacity of ACE2-WT and ACE2-QMP to block cellular infection of F) Wuhan SARS-CoV-2 spike-pseudotyped lentivirus expressing GFP to 293T-ACE2-TMPRSS2 cells, and G) live virus to Vero E6 cells, in comparison with CD147-QMP (mean ± SD, *n* = 2). H) Illustrations of the albumin-fused dimeric ACE2 (residues 18–740; 300 kDa) compared with monomeric ACE2 lacking CLD (residues 18–615; 136 kDa). I) Representative analytical SEC profile of dimeric and monomeric ACE2-albumin fusions. J) ELISA results showing the binding of dimeric and monomeric ACE2-albumin fusions to Wuhan SARS-CoV-2 spike (mean ± SD, *n* = 2). K) Representative pseudovirus neutralization and L) live virus neutralization experiments of monomeric ACE2(18–615)-QMP and dimeric ACE2-QMP (mean ± SD, *n* = 4 or 2, respectively). Unpaired two-tailed *t*-test was used for statistical analysis, where *ns* = not significant, **P = 0.0043, and ****P < 0.0001. The illustrations were created with BioRender.com.

SARS-CoV-2 is characterized by its spike protein, which is composed of two subunits, S1 and S2 (Fig. 2A) (15), in which S1 contains the RBD responsible for binding to ACE2 on human cells. Both ACE2-WT and ACE2-QMP were shown to bind strongly and equally well to the recombinant spike protein of the ancestral SARS-CoV-2 strain (Wuhan) and its derived RBD in ELISA (Fig. 2B–D). SPR-derived binding kinetics revealed nanomolar affinity for both ACE2-albumin fusions and unfused ACE2, in line with previously reported data (57), whereas human albumin did not bind (Fig. S2; Table S2). Interestingly, an albumin fusion containing the previously postulated SARS-CoV-2 receptor CD147 (CD147-QMP) (22) did not show detectable binding (Fig. 2C and D), supported by later published studies (23, 24). Next, we tested the ability of ACE2-albumin fusions to block cellular SARS-CoV-2 infection (Fig. 2E) of 293T cells by Wuhan spike-pseudotyped lentivirus expressing GFP (81). The 293T cells were transfected to express both the transmembrane-bound human ACE2 and TMPRSS2 (293T-ACE2-TMPRSS2). Both ACE2-WT and ACE2-QMP effectively blocked viral infection with a half-maximal inhibitory concentration (IC_50_) of 15–20 nM, which was about 2- to 4-fold higher than that of unfused ACE2 (7.9 nM) (Figs. 2F and S2; Table S3). This was also the case when live SARS-CoV-2 Wuhan was exposed to the kidney epithelial Vero E6 cells, displaying an IC_50_ of 250–300 nM (Fig. 2G; Table S3). Consistent with the binding data, neither CD147-QMP (Fig. 2F and G; Table S3) nor human albumin (Fig. S3; Table S3) was able to suppress viral infection.

### The dimeric nature of ACE2 is required for efficient blockage of viral infection

Several reports have revealed that soluble monomeric ACE2 exhibits lower binding affinity to SARS-CoV-2 spike and is less potent in viral neutralization when compared with dimeric formats (52, 58). To address if dimerization of albumin-fused ACE2 was required for effective blockage of SARS-CoV-2 infection, an alternative design was made by excluding the CLD of ACE2 (Figs. 2H and S3) (16). This resulted in well-produced monomeric fusion formats, yielding pure, soluble ACE2(18–615)-WT and ACE2(18–615)-QMP with expected MW (136 kDa) (Figs. 2I and S3). Similar to the dimeric fusions, monomeric ACE2(18–615)-QMP showed a greater pH-dependent binding to human FcRn than ACE2(18–615)-WT (Fig. S3), which also demonstrated more efficient recycling (Fig. S3) and transcytosis (Fig. S3) *in vitro*. This resulted in a 1.6-fold extended plasma half-life in human FcRn Tg32 mice upon IV administration (Fig. S3), and a 2.1-fold increase in transcytosis across selective mucosal epithelium following IN delivery in the transgenic mice (Fig. S3). However, their ability to engage the Wuhan SARS-CoV-2 spike and RBD was severely reduced, when compared with the dimeric counterparts in ELISA (Figs. 2J and S4). SPR-derived binding kinetics revealed that the affinity of both monomeric unfused ACE2(18–615) and ACE2(18–615)-albumin fusions toward the SARS-CoV-2 RBD was up to 363-fold lower than that of the dimeric formats (Fig. S4; Table S2). In line with this, monomeric ACE2(18–615) did not exhibit inhibition potency when compared with the dimeric ACE2 formats (Fig. S4; Table S3). As such, the monomeric ACE2(18–615)-albumin fusions poorly blocked cellular infection of both SARS-CoV-2 pseudovirus and live virus (Figs. 2K, L and S4; Table S3). Hence, the CLD of ACE2 required for dimerization is important for efficient blockage of viral infection.

### ACE2-YTY-QMP potently blocks a range of SARS-CoV-2 mutant variants

**Fig. 3.**
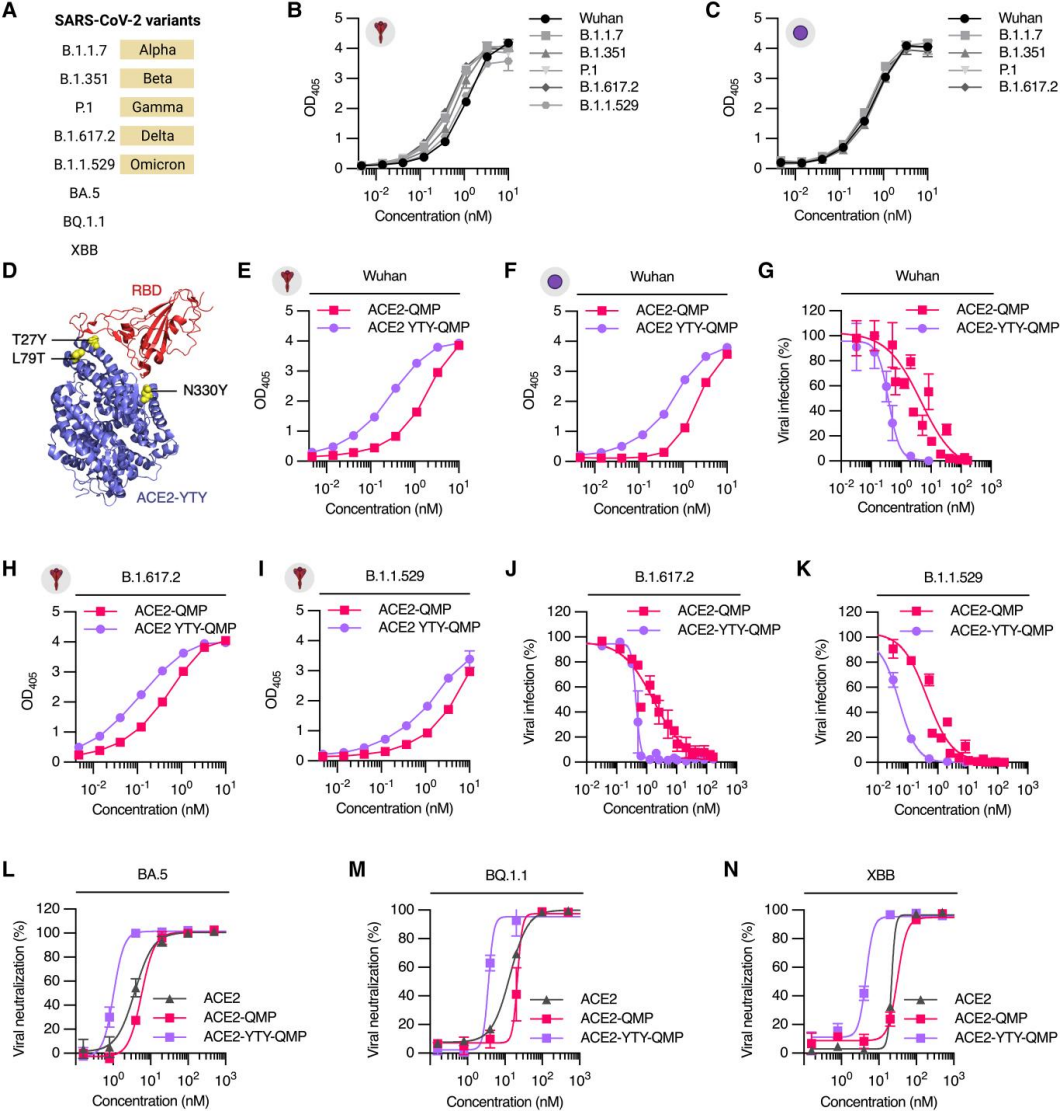
Engineered ACE2-YTY fused albumin potently blocks cellular infection against SARS-CoV-2 mutant variants. A) Overview of SARS-CoV-2 mutant variants used in the study. B and C) Representative ELISA results showing the binding of dimeric ACE2-QMP to recombinant B) spike and C) derived RBD of SARS-CoV-2 variants (mean ± SD, *n* = 2). D) Crystallographic illustration of the YTY-containing ACE2 bound to SARS-CoV-2 RBD. YTY mutations (T27Y, L79T, and N330Y) are shown as yellow spheres. The figure was made using PyMOL with the crystal structure data deposited (PDB ID: 7U0N). E and F) Representative ELISA results showing the binding of QMP albumin-fused dimeric ACE2 and ACE2-YTY to recombinant E) spike and F) RBD of the Wuhan SARS-CoV-2 (mean ± SD, *n* = 2). G) Pseudovirus neutralization experiment showing the capacity of dimeric ACE2-QMP and ACE2-YTY-QMP to block cellular infection of Wuhan spike-pseudotyped lentivirus to 293T-ACE2-TMPRSS2 cells (mean ± SD, *n* = 2, 3 independent experiments). H and I) Representative ELISA results showing the binding of ACE2-QMP and ACE2-YTY-QMP to recombinant spike of the H) B.1.617.2 and (I) B.1.529 variants. J and K) Pseudovirus neutralization experiments of dimeric ACE2-YTY-QMP against the J) B.1.617.2 and K) B.1.1.529 variants, in comparison with ACE2-QMP (mean ± SD, *n* = 2, 2 independent experiments). L–N) Representative live virus neutralization experiments of dimeric ACE2-YTY-QMP against the Omicron subvariants L) BA.5, M) BQ.1.1, and N) XBB, in comparison with unfused ACE2 and ACE2-QMP (mean ± SD, *n* = 3). The illustrations were created with BioRender.com.

As ACE2 is the principal host receptor required for viral entry, we confirmed that the dimeric ACE2-QMP binds strongly to recombinant SARS-CoV-2 spike and derived RBD of the B.1.1.7 (Alpha), B.1.351 (Beta), P.1 (Gamma), B.1.617.2 (Delta), B.1.1.529 (Omicron) variants (Fig. 3A–C). Furthermore, to enhance the ability of the dimeric design to block viral infection, we took advantage of an engineered ACE2 variant with three amino acid substitutions (YTY) (Fig. 3D). When combined with QMP albumin, the designed ACE2-YTY-QMP fusion was well produced and demonstrated similar human FcRn binding and cellular transport properties to those of ACE2-QMP (Fig. S5). Furthermore, incorporation of the YTY amino acid substitutions in ACE2 enhanced engagement of Wuhan SARS-CoV-2 spike and RBD by about 10-fold (Figs. 3E, F and S5; Table S2), which translated into 12-fold more efficient blockage of the corresponding spike-pseudotyped lentivirus than ACE2-QMP (Fig. 3G; Table S4), as well as live Wuhan SARS-CoV-2 (Fig. S5; Table S5). Notably, ACE2-YTY-QMP also showed enhanced binding to the SARS-CoV-2 spike protein and derived RBD from the B.1.617.2 and B.1.1.529 variants, compared with those lacking mutations (Figs. 3H, I and S6; Table S2). This gave 3.8- and 8.8-fold more efficient blockage of cellular infection with the corresponding spike-pseudotyped lentiviruses than ACE2-QMP, respectively (Fig. 3J and K; Table S4). Other more recent subvariants of Omicron include BA.5, BQ.1.1, and XBB (Fig. 3A). Importantly, the ACE2-albumin fusions demonstrated effective neutralization against these variants, in which the YTY-containing ACE2 fusion showed up to 7.6-fold improved efficacy against the Omicron subvariants in comparison with ACE2-QMP, as well as unfused ACE2 (Fig. 3L–N; Table S6). Accordingly, these findings demonstrate the broad-spectrum activity of ACE2-YTY-QMP against multiple variants of SARS-CoV-2.

## Discussion

The low effectiveness of COVID-19 vaccines in specific patient groups, such as immunocompromised patients (5–8), along with the emergence of SARS-CoV-2 variants capable of evading vaccine-induced immunity (9–12), underlines the need for novel treatment approaches. In this regard, the clinical trials of APN01, a recombinant, soluble truncated dimeric human ACE2, have revealed a potential therapeutic target with no severe undesirable side reactions (40, 44–48). Moreover, as the virus continues to evolve, developed anti-spike monoclonal antibodies may lose their neutralizing efficacy against new escape mutant variants, as recently observed with the Omicron lineages (26–31). In contrast, APN01, alongside other recombinant ACE2 formats, has been reported to be effective in blocking several SARS-CoV-2 variants (39–42). Moreover, such recombinant ACE2 presents an attractive candidate for the treatment of a range of conditions beyond that of COVID-19 (45, 82–84). This motivates in-depth studies on the potential of therapeutic human ACE2 combined with strategies that can improve its pharmacokinetic properties and routes of administration.

Here, we report on an albumin-based strategy for extending the plasma half-life of soluble truncated dimeric human ACE2, based on the design of the clinical-grade APN01, as demonstrated in state-of-the-art human FcRn-expressing mice. This was achieved by fusing the extracellular domains of dimeric ACE2 (residues 18–740) to human albumin. In line with findings from a recent study (57), the fusion of ACE2 with WT albumin extends its plasma half-life *in vivo*. Our results demonstrated that the ACE2-WT fusion increased the half-life by about 2.3-fold compared with unfused ACE2 in transgenic mice expressing human FcRn. To further improve the pharmacokinetics of the ACE2-albumin fusion format, we took advantage of an engineered full-length human albumin variant containing three amino acid substitutions (QMP) (73). In comparison with WT human albumin, the QMP-containing albumin displays a superior ability to engage human FcRn in a pH-dependent manner (73). In accordance with published reports (73, 77), the QMP albumin-fused dimeric ACE2 resulted in a 2.4-fold improved plasma half-life when compared with the WT counterpart. However, IV treatment may not always be the most optimal route of administration, in which high drug concentrations must be achieved in the blood to effectively reach the sites of infection, such as the lungs and the gut. In our present work, we show that the ACE2-QMP design can also be given subcutaneously, which efficiently reached the blood in high amounts. Another favorable feature is the ability of QMP albumin to enhance transmucosal transport of ACE2 across selective pulmonary epithelial barriers upon IN delivery in a needle-free manner. This suggests that the ACE2-QMP fusion design can provide its mode of action both locally and systemically, making it highly relevant in the context of respiratory infectious diseases like COVID-19, where the lungs serve as the primary site of SARS-CoV-2 infection (2).

When employing human albumin as a fusion carrier, it is crucial to consider the human form of FcRn throughout the study, in both *in vitro* and *in vivo* settings. Due to cross-species differences, human albumin binds poorly to mouse FcRn and, thereby, is not rescued from intracellular degradation in conventional mice expressing the mouse receptor (85, 86). However, the human FcRn-expressing Tg32 mice continuously produce endogenous mouse albumin, which binds to the human receptor more strongly than human albumin (87). Consequently, the high concentration of mouse albumin will compete with injected human albumin for binding to human FcRn, creating a greater competitive environment than in the presence of endogenous human albumin. Despite this strong competitive pressure, QMP albumin-fused ACE2 is more favorably rescued from intracellular degradation than the WT counterpart, as well as having enhanced transport across the selective respiratory mucosal barriers (73). As albumin is the most abundant protein in blood (72), the QMP albumin ensures the effective bioavailability of its fusion partner, even amid a prevalent presence of endogenous albumin *in vivo*.

To advance the design of potent soluble ACE2 candidates, engineered ACE2 variants with amino acid substitutions that enhance their affinity for viral spike proteins have been developed (42, 52, 53, 67, 68). In this study, we explored the use of one such promising candidate, namely YTY, which has shown to be effective against multiple SARS-CoV-2 variants (52, 53). Our data support that YTY-containing ACE2, in the context of a dimeric fusion with the QMP albumin variant, demonstrates improved viral binding and blockage of cellular infection against Delta (B.1.617.2) and Omicron (B.1.1.529, BA.5, BQ.1.1, and XBB) variants, in comparison with WT ACE2. As such, engineered ACE2 holds promise as it can potentially combat future SARS-CoV-2 variants and offer an appealing alternative to anti-spike monoclonal antibodies, which often encounter high viral resistance from emerging mutations like Omicron subvariants (26–31). As neutralizing monoclonal antibodies designed for COVID-19 treatment are generally targeted toward distinct regions of the viral spike protein, the emergence of new SARS-CoV-2 variants with mutated spike proteins may obstruct antibody recognition and binding, thereby diminishing their potency (4). Nevertheless, to increase the therapeutic benefits, there is room for exploring combinational therapies, such as monoclonal antibodies in conjunction with soluble recombinant ACE2 (88). As a major drawback of soluble ACE2 is its extremely short plasma half-life (44), it would be intriguing to combine such strategies with QMP-containing albumin to provide improved pharmacokinetics of ACE2.

An alternative solution could entail the fusion of ACE2 with the constant Fc of IgG1 or other subclasses (51–56), leveraging FcRn-mediated cellular recycling (51, 53). However, this would result in a dimeric ACE2-Fc fusion, in which dimerization is forced by the homodimeric nature of both ACE2 and the hinge-coupled Fc. This is in contrast to ACE2-albumin fusions, which naturally assemble into a homodimer via the dimerizing domains of ACE2 (16). Moreover, the Fc fragment is taken out of its context of a full-length IgG1 format. As recent reports have underlined a key role of the fragment-antigen binding regions of IgG antibodies on pharmacokinetics (76, 89), this may explain why Fc fusions have a shorter plasma half-life than full-length IgG1 antibodies. In the case of fusion to human albumin, the whole albumin molecule is used as a carrier, where optimal human FcRn binding can be secured and promoted by the QMP amino acid substitutions. Importantly, albumin is a simple single nonglycosylated polypeptide that is effector negative (72). In contrast, IgG1 Fc can engage a range of Fc receptors and complement factor C1q, which may activate unwanted immune reactions (55, 90). A way to avoid such cytotoxic effects involves Fc engineering to eliminate the ability to engage Fc-binding molecules, except for FcRn (91).

Another strategy is the fusion of ACE2 with a bacteria-derived ABD (58–60), which is based on the principle of indirectly targeting FcRn. The ACE2-fused ABD is designed to interact with endogenous albumin, thereby conferring the ability to engage FcRn and evade lysosomal intracellular degradation, as demonstrated for other fusion products (92). In conventional mice, ACE2-ABD exhibited a half-life of 1 day (58), comparable with our ACE2 fusion with WT albumin, however still shorter than that of ACE2-fused QMP albumin with a half-life of 2.6 days. While the use of ABD is an attractive half-life extension approach due to the abundance of albumin (72), it can also be engineered for improved stability and albumin binding affinity (93), and importantly, to reduce or abolish undesired immunogenicity, as demonstrated upon repeated administrations in mice, rats, and humans (93–95). Notably, it can also be administered intranasally, as recently shown for ACE2-ABD in mice (60).

An FcRn-independent approach is the fusion of CTB to full-length ACE2 (41, 63–66). CTB is a well-established transmucosal carrier for oral protein delivery, which has been used to treat conditions such as diabetic retinopathy (64) and pulmonary arterial hypertension (63, 66). The principle is based on that CTB has a high affinity for the GM1 ganglioside, a glycolipid found on the surface membrane of various cell types, including gastrointestinal epithelial cells (88). Here, GM1 acts as the cellular receptor for CTB, as well as other toxins and pathogens, thereby facilitating cellular internalization. Interestingly, studies have suggested that GM1 ganglioside may play a role as a receptor for the entry of SARS-CoV-2 into human cells, alongside ACE2 and other receptors and molecules (20, 21). As such, oral administration of CTB-ACE2 results in transmucosal transport and inhibition of SARS-CoV-2 internalization, either by direct blockage of the viral receptor-binding sites or by saturating both receptors on the surface of epithelial cells (41, 65). When full-length ACE2 fused with CTB was expressed in plant and provided in a chewing gum, it demonstrated effective reduction in viral infection and transmission against the Delta and Omicron variants of SARS-CoV-2 (41). A combination strategy and comparison between the CTB-based design and that of engineered albumin would be an intriguing area for follow-up studies.

Lastly, non-invasive methods of administration, such as nasal and oral delivery, may improve patient compliance and convenience (96). Yet, both approaches pose their challenges. While nasal administration of albumin-fused ACE2 is motivated by delivery at the primary site of infection, combined with the ability to engage FcRn for half-life extension and transmucosal transport to local tissues and blood (76), bioencapsulation may not be required to withstand the acidic and enzymatic conditions within the gastrointestinal tract. On the other hand, oral administration may provide more efficient uptake due to the large absorption surface area of the gut (96, 97), and biologics may be protected by bioencapsulation, for instance through the use of plant cells (98). Considering the high viral loads in saliva of SARS-CoV-2-infected individuals, oral delivery of ACE2 may have the potential to alleviate viral transmission (61, 62). Hence, selecting the appropriate delivery route depends on several factors, including the characteristics of the biologics, the target sites of action, the desired pharmacokinetic profile, and patient acceptability.

## Conclusion

Taken together, our study shows that soluble truncated dimeric human ACE2 can be tailored for favorable pharmacokinetic properties and efficient needle-free delivery across mucosal barriers by fusion to engineered human albumin with improved pH-dependent engagement to human FcRn. This strategy may increase the prophylactic and therapeutic utility of recombinant ACE2 while also enhancing patient compliance and convenience. Additionally, combining this concept with engineered ACE2 demonstrating greater affinity for the virus is likely to be pan-SARS-CoV-2 specific and retain the ability to neutralize a wide range of immune escape variants, which is an important attribute considering the variable prevalence of viral mutations worldwide and potential future outbreaks. Notably, the QMP albumin-based strategy offers promising research and clinical development prospects, which extends beyond COVID-19 and offers innovative frameworks for a range of diseases that require long-acting, non-invasive mucosal delivery.

## Materials and methods

### Vector design and protein production of albumin-fused ACE2

Complementary DNA (cDNA) sequences encoding human albumin-fused ACE2 (Table S7) were synthesized and subcloned into pFUSE2ss-CLIg-hk (InvivoGen) using the restriction enzymes *EcoRI* and *NheI*, in which the sequence corresponding to the N-terminal end of truncated ACE2 was fused with cDNA encoding full-length WT human albumin or an engineered human albumin variant (E505Q/T527M/K573P; QMP) (GenScript) (73).

The constructed vectors were transiently transfected into Expi293F suspension cells using ExpiFectamine 293 Transfection Kit (Gibco), according to the manufacturer's instruction. In brief, a mixture of vector DNA and ExpiFectamine 293 Reagent, diluted in Opti-MEM I Reduced Serum Medium, was added dropwise to Expi293F cells (3 × 10^6^ viable cells/mL, > 95% cell viability) cultured in Expi293 Expression Medium. The transfected cells were incubated at 37 °C with 80% relative humidity and 8% CO_2_ on an orbital shaker platform set to 125 rpm (Thermo Scientific). Transfection enhancers were added to the cell cultures 16–20 h post-transfection. After 4 days of incubation, the cell culture media were harvested by centrifugation at 290–400 × *g* for 20 min.

The secreted albumin-fused ACE2 was purified on a CaptureSelect Human Albumin Affinity Matrix (Thermo Scientific), packed in a 5-mL OPUS Chromatography Column (Repligen), and eluted with a solution of 2 M MgCl_2_ and 20 mM Tris (pH 7.0). Following elution, the proteins were buffer-exchanged to 1X phosphate-buffered saline (PBS; pH 7.4; Sigma-Aldrich) and up-concentrated using Amicon Ultra-15 Centrifugal Filter Units with a cutoff of 50 kDa (Merck Millipore). SEC was performed on an ÄKTA avant 150 Chromatography System (Cytiva) with a Superdex 200 Increase 10/300 GL Column (Cytiva) to acquire pure soluble fractions of ACE2-albumin fusions. Dimerization of albumin-fused ACE2 variants was determined by analytical SEC on a Superdex 200 Increase 3.2/300 Column (Cytiva), together with full-length human albumin and IgG1 as size references.

### Vector design and protein production of SARS-CoV-2 spike and derived RBD

cDNA encoding 8×His-tagged HexaPro spike protein (99) and 6×His-tagged RBD (100) of SARS-CoV-2, Wuhan, in the expression plasmids pαH and pCAGGS, respectively, were kindly provided by Juni Andréll (Stockholm University, Sweden). Mutant SARS-CoV-2 spike and derived RBD variants were made by site-directed mutagenesis using the DNA vectors of 8×His-tagged HexaPro spike protein and 6×His-tagged RBD (GenScript) as templates, respectively (Table S8).

The constructed vectors were transiently transfected into Expi293F suspension cells using ExpiFectamine 293 Transfection Kit (Gibco), as described above. The secreted His-tagged spike and RBD variants were purified on HisTrap HP 5 mL Prepacked Columns (Cytiva) and eluted with 250 mM imidazole (Sigma-Aldrich) in PBS (pH 7.4). Following elution, the proteins were buffer-exchanged to PBS (pH 7.4) and up-concentrated using Amicon Ultra Centrifugal Filter Units with a cutoff of 50 or 10 kDa (Merck Millipore) for spike and RBD, respectively. SEC was then performed on an ÄKTA avant 150 Chromatography System (Cytiva) with a Superdex 200 Increase 10/300 GL Column (Cytiva) to acquire pure soluble fractions of proteins.

### Production of human FcRn

Soluble His-tagged human FcRn (human FcRn-His) was synthesized, as previously described (101, 102). The viral stock was generously provided by Prof. Sally Ward (University of Southampton, United Kingdom).

### Protein verification

Protein concentrations were determined using a DS-11+ (M/C) Spectrophotometer (DeNovix). Protein purity and molecular size were verified with nonreducing SDS-PAGE on a Bolt 12% Bis-Tris Plus Gel (Invitrogen). In brief, 2 µg of protein samples were mixed with 4× Bolt LDS Sample Buffer (Invitrogen) and Milli-Q water, according to the manufacturer's instruction. The gel was run in 1× Bolt MES SDS Running Buffer (Invitrogen) at 200 V for 22 min and stained with Bio-Safe Coomassie Staining Solution (Bio-Rad) for 25–45 min with moderate agitation, before being destained in water overnight until the background became clear. Spectra Multicolor Broad Range Protein Ladder (10–260 kDa; Thermo Scientific) was used for monitoring protein migration during SDS–PAGE, as well as sizing of proteins.

### Binding to human FcRn

In ELISA, 96-well EIA/RIA Clear Flat Bottom Polystyrene Microplates (Corning) were coated with 8 µg/mL (100 µL/well) of a recombinant Fc-engineered human IgG1 mutant (M252Y/S254T/T256E/H433K/N434F; MST/HN), with specificity for 4-hydroxy-3-iodo-5-nitrophenylactic acid (103), diluted in PBS (pH 7.4), overnight at 4 °C. The wells were blocked with 250 μL of PBS containing 4% (w/v) skimmed milk powder (Sigma-Aldrich) (PBS/M; pH 7.4) for 1 h at room temperature, then washed 3 times with 250 μL of PBS containing 0.05% (v/v) Tween 20 (Sigma-Aldrich) (PBS/T; pH 5.5 or 7.4). Then, 10 µg/mL (100 µL/well) of human FcRn-His in PBS/T/M (pH 5.5 or 7.4) were added and incubated for 1 h at room temperature, before washing as indicated above. Subsequently, serial dilutions (100 µL/well) of albumin-fused ACE2 or unfused ACE2 (75–0.03 nM) in PBS/T/M (pH 5.5 or 7.4) were added for 1 h at room temperature. Following washing, 125 ng/mL (100 µL/well) of Goat anti-Human Albumin Cross-Adsorbed Antibody ALP-Conjugated (Bethyl Laboratories, Inc.) in PBS/T/M (pH 5.5 or 7.4) was added for 1 h at room temperature, washed, and followed by 1 mg/mL (100 µL/well) of *p*-Nitrophenyl Phosphate Substrate (Sigma-Aldrich) dissolved in diethanolamine buffer (pH 9.8). Absorbance was measured at 405 nm using a Sunrise Absorbance Reader (TECAN).

To analyze binding kinetics, SPR was performed using a Biacore T200 instrument (Cytiva). As running and dilution buffer solutions, 67 mM phosphate buffer containing 0.15 M NaCl and 0.005% (v/v) Tween 20 (pH 5.5 or 7.4) was used. Series S CM5 Sensor Chips (Cytiva) were covalently immobilized with 15 μg/mL of albumin-fused ACE2, using an Amine Coupling Kit (Cytiva), according to the manufacturer's instruction. Kinetic measurements were done by injecting 2-fold serial dilutions of monomeric human FcRn-His (275–5.9 nM for WT albumin fusions and 125–2 nM for QMP albumin fusions) in duplicates over immobilized albumin-fused ACE2 at a flow rate of 30 μL/min at 25 °C for 120 s during the association phase, followed by dissociation over 600 s. After each run, the chip surface was regenerated using the phosphatase buffer (pH 7.4) at a flow rate of 30 μL/min with a contact time of 120 s and a stabilization period of 10 s. Kinetic rate values were determined using 1:1 Langmuir binding model in the Biacore T200 Evaluation V3.0 Software 9 (Cytiva). Binding responses from control CM5 flow cells and blank injections (references) were subtracted from each interaction curve to adjust for nonspecific binding and bulk buffer effects.

### Direct albumin sandwich ELISA

A two-way antihuman albumin ELISA setup with solutions of known concentration (standards) was used to quantify the amounts of albumin-fused ACE2 present in the media collected from the *in vitro* cellular recycling and transcytotic studies, or in the plasma samples collected from *in vivo* studies. In brief, 96-well EIA/RIA Clear Flat Bottom Polystyrene Microplates (Corning) were coated with 1 µg/mL (100 μL/well) of Polyclonal Anti-Human Albumin antibody from goat (Sigma-Aldrich) diluted in PBS (pH 7.4) for HERA and Transwell assay samples, or with Monoclonal Anti-Human Serum Albumin from mouse (Abcam) for plasma samples, overnight at 4°C. The wells were blocked with 200 μL of PBS/M (pH 7.4) for 1 h at room temperature, followed by three washes with 250 μL of PBS/T (pH 7.4). Subsequently, samples and standards prepared in PBS/T/M (pH 7.4) were added for 1 h at room temperature, and washed as described above. Samples collected from HERA and Transwell assay were diluted 1:2 in 2-fold serial dilutions (100 µL/well), or 1:50 dilution in 3-fold serial dilutions (50 µL/well) for plasma samples. Captured albumin-fused ACE2 was detected by adding 125 ng/mL (100 µL/well) of Goat anti-Human Albumin Cross-Adsorbed Antibody ALP-Conjugated (Bethyl Laboratories, Inc.) in PBS/T/M (pH 7.4) for 1 h at room temperature, washed, and followed by 1 mg/mL (100 µL/well) of *p*-Nitrophenyl Phosphate Substrate (Sigma-Aldrich) dissolved in diethanolamine buffer (pH 9.8). Absorbance was measured at 405 nm (with or without reference wavelength at 620 nm) using a Sunrise Absorbance Reader (TECAN).

### Human endothelial cellular recycling assay

Cellular recycling was studied using HMEC-1 cells stably expressing HA-human FcRn-EGFP in HERA (78), as previously described (75). HMEC-1-FcRn cells were cultured in MCDB 131 Medium (Gibco) supplemented with 10% heat-inactivated Fetal Bovine Serum (FBS; Sigma-Aldrich), 1× Penicillin–Streptomycin (Gibco), 2 mM L-Glutamine (Gibco), 10 ng/mL Recombinant Mouse Epidermal Growth Factor (EGF; Gibco), 1 µg/mL Hydrocortisone (Sigma-Aldrich), 100 µg/mL Geneticin Selective Antibiotic (Gibco), and 5 µg/mL Blasticidin S HCl (Gibco), at 37°C in a humidified atmosphere of 5% CO_2_. In brief, HMEC-1-FcRn cells were seeded at a density of 3.75 × 10^4^ cells/well in a Costar 24-well TC-treated Multiple Well Plate (Corning). After 20 h of incubation, the cells were washed 3 times with prewarmed Hank's Balanced Salt Solution (HBSS; Gibco) and starved for 1 h. Subsequently, 800 nM (250 µL/well) of albumin-fused ACE2 or unfused ACE2, diluted in HBSS, were added and incubated for 3 h. The cells were then washed 5 times with ice cold HBSS, followed 3-h incubation in 225 µL of assay medium (complete culture medium: without FBS, Geneticin and Blasticidin S HCL, and supplemented with 1X Eagle's Minimum Essential Medium Non-Essential Amino Acids Solution [Gibco]). The assay media were collected, and the amounts of recycled albumin-fused ACE2 were quantified by direct albumin sandwich ELISA, as described above.

### Transwell assay

Transcytosis was studied using the MDCKII cells stably express human FcRn/β2-microglobulin in a Transwell assay. MDCKII-FcRn cells were cultured in Dulbecco's Modified Eagle Medium (DMEM) with GlutaMAX Supplement (Gibco), containing 10% heat-inactivated FBS (Sigma-Aldrich) and 300 µg/mL Geneticin Selective Antibiotic (Gibco), at 37 °C in a humidified atmosphere of 5% CO_2_. In brief, MDCKII-FcRn cells were seeded at a density of 1.25 × 10^6^ cells/well in 12-mm Transwell-COL Collagen-coated 0.4 µm Pore PTFE Membrane Inserts (Corning) in Costar 12-well TC-treated Multiple Well Plates (Corning), after overnight equilibration with complete culture medium. Following 24 h of incubation, transepithelial electrical resistance (600–1,100 Ω cm^2^) was measured using a Millicell ERS-2 Voltohmmeter (Millipore). The cells were rinsed 3 times with prewarmed HBSS (Gibco) and starved for 1 h. Subsequently, 800 nM (200 µL/well) of albumin-fused ACE2 and unfused ACE2, diluted in HBSS, were added to the Transwell inserts (apical side), and 900 µL of the incubation solutions were collected from the wells (basolateral side) after 4 h of sample incubation. The amounts of transcytosed protein were quantified by direct albumin sandwich ELISA, as described above.

### Plasma half-life

Half-life studies of albumin-fused ACE2 and unfused ACE2 were performed in homozygous or hemizygous human FcRn Tg32 mice (B6.Cg-*Fcgrt^tm1Dcr^* Tg(FCGRT)32Dcr/DcrJ, The Jackson Laboratory). In homozygous Tg32 mice, groups of female and male mice (6–11 weeks, 5 mice per group) were given IV injection of 2 mg/kg of albumin-fused ACE2 variants or 1.16 mg/kg of unfused ACE2, diluted in PBS (pH 7.4). Blood (25 μL) was withdrawn from the saphenous vein on days 1, 2, 3, 4, 5, and 7 post-injection for albumin-fused ACE2, and after 4, 6, 9, 12, 20, 28, and 48 h for unfused ACE2, using microcapillary pipettes (Hirschmann). Plasma was isolated by centrifugation at 17,000 × *g* for 10–15 min at 4 °C and stored at −20 °C until analysis. This study was carried out at the Section of Comparative Medicine, Rikshospitalet, Oslo University Hospital, Norway, and was approved by the Norwegian Food Safety Authority (FOTS ID 26082). In hemizygous Tg32 mice, groups of male mice (7–8 weeks, 5 mice per group) were given either IV or SC injection of 2 mg/kg of albumin-fused ACE2. Blood (25 μL) was withdrawn from the retro-orbital sinus on days 1, 2, 3, 4, 5, 7, 10, and 12 (for IV administration) or 14 (for SC administration) post-injection. Blood samples were mixed with 1 μL of 1% K3-EDTA to prevent coagulation before plasma was isolated by centrifugation at 17,000 × *g* for 5 min at 4°C and diluted 1:10 in 50% glycerol buffer in PBS (pH 7.4) for storage. These studies were carried out at The Jackson Laboratory . The amounts of protein in plasma samples were quantified using direct albumin sandwich ELISA, as described above.

Plasma concentrations were presented as percentages remaining in the blood at different post-injection time points relative to the initial blood sampling (set as 100%), day 1 for albumin-fused ACE2, and 4 h for unfused ACE2. Regression analyses for curve fitting were performed using GraphPad Prism 9 Software (Version 9.0.2; GraphPad Software, Inc.). Linear regression analysis was applied to ACE2-albumin fusions. The elimination half-life was calculated using the following formula:


$$t_{1/2\beta }=\log\;\frac{0.5}{A_{e}/A_{0}}\times t,$$

where $t_{1/2\beta }$ is the plasma half-life evaluated during the beta phase, *A_e_* is the remaining concentration, *A*_0_ is the concentration on day 1, and *t* is the elapsed time. For unfused ACE2, a two-phase exponential decay model in nonlinear regression analysis was used for data fitting and half-life calculation.

### Pulmonary delivery

Mucosal delivery of albumin-fused ACE2 upon IN administration was studied in homozygous human FcRn Tg32 mice (B6.Cg-*Fcgrt^tm1Dcr^* Tg(FCGRT)32Dcr/DcrJ, The Jackson Laboratory). Groups of female and male mice (6–8 weeks, 5 mice per group) were anesthetized by intraperitoneal injection of ZRF (Zoletil Forte, Fentanyl, Rompun, and Sodium Chloride; 0.1 mL/10 g). When sedated, 100 μg (10 μL) of albumin-fused ACE2 were given to each nostril. Blood (25 μL) was withdrawn from the saphenous vein after 4, 8, 48, and 96 h using microcapillary pipettes (Hirschmann). Plasma was isolated by centrifugation at 17,000 × *g* for 10–15 min at 4 °C and stored at −20 °C until analysis. The amounts of protein in plasma samples were quantified using direct albumin sandwich ELISA, as described above. The study was carried out at the Section of Comparative Medicine, Rikshospitalet, Oslo University Hospital, Norway, and was approved by the Norwegian Food Safety Authority (FOTS ID 26082).

### Binding to SARS-CoV-2 spike and derived RBD

In ELISA, 96-well EIA/RIA Clear Flat Bottom Polystyrene Microplates (Corning) were coated with 40 nM (100 μL/well) of SARS-CoV-2 (2019-nCoV) Spike S1-His Recombinant Protein (Sino Biological) or 6×His-tagged RBD, or serial dilutions of spike and derived RBD mutants (30–0.3 nM), diluted in PBS (pH 7.4) overnight at 4 °C. Following block and wash, as previously described, albumin-fused ACE2 in PBS/T/M (pH 7.4) were added in 3-fold serial dilutions (150.4–0.07 nM) or at a fixed concentration of 7.5 nM for 1 h at room temperature. Following washing, captured albumin-fused ACE2 was detected by adding 125 ng/mL (100 µL/well) of Goat anti-Human Albumin Cross-Adsorbed Antibody ALP-Conjugated (Bethyl Laboratories, Inc.) in PBS/T/M (pH 7.4) for 1 h at room temperature, washed, and followed by 1 mg/mL (100 µL/well) of *p*-Nitrophenyl Phosphate Substrate (Sigma-Aldrich) dissolved in diethanolamine buffer (pH 9.8). Absorbance was measured at 405 nm, with 620 nm as reference wavelength, using a Sunrise Absorbance Reader (TECAN).

To analyze binding kinetics, SPR was performed using a Biacore T200 instrument (Cytiva). 1× HBS-P + Buffer (Cytiva) was used as running and dilution buffer solutions. Series S CM5 Sensor Chips (Cytiva) were covalently immobilized with 5 μg/mL of monomeric RBD derived from SARS-CoV-2 from Wuhan or B.1.617.2 (∼400 RU), using an Amine Coupling Kit (Cytiva) according to the manufacturer's instruction. Kinetic measurement was evaluated by injecting 2-fold serial dilutions of albumin-fused ACE2 (25–0.78 nM) in duplicates over immobilized RBD at a flow rate of 30 μL/min at 25 °C for 180 s during the association phase, followed by dissociation over 1,770 s for strong binders or 600 s for weak binders. After each run, the chip surface was regenerated using 10 mM Glycine-HCl (Cytiva; pH 2.5) at a flow rate of 30 μL/min with a contact time of 30 s and a stabilization period of 10 s. Kinetic rate values were determined using 1:1 Langmuir binding model in the Biacore T200 Evaluation V3.0 Software (Cytiva). Binding responses from control CM5 flow cells and blank injections (references) were subtracted from each interaction curve to adjust for nonspecific binding and bulk buffer effects.

### Pseudovirus neutralization (Wuhan, B.1.617.2, and B.1.1.529)

Neutralization assay was performed using replication deficient human SARS-CoV-2 spike-pseudotyped HIV-1 produced in WT human embryonic kidney-derived 293T cells expressing both human ACE2 and TMPRSS2, as previously described (81). In brief, 293T-ACE2-TMPRSS2 cells were cotransfected with 1 μg of plasmid encoding prototypic SARS-CoV-2 spike protein (pCAGGS-Spike Δc19), 1 μg of pCMV-Gag-Pol Retroviral Vector, and 1.5 μg of GFP-encoding plasmid (CSGW). At 48- and 74-h post-transfection, viral supernatants were filtered through a 0.45-μm syringe filter, pelleted by centrifugation at 28,000 × *g* for 2 h, and then resuspended in DMEM (Gibco). To test the ability of albumin-fused ACE2 to block cellular infection, 293T-ACE2-TMPRSS2 cells were seeded at a density of 0.75 × 10^3^ cells/well in 96-well plates and allowed to adhere overnight. Prior to the experiment, 20 μL of spike-pseudotyped HIV-1 was mixed with serial dilutions of albumin-fused ACE2 variants or QMP albumin-fused CD147 (200–0.15  μg/mL) and incubated for 40 min at room temperature, before transferring to the cultured cells (10 μL/well). Viral infection was evaluated 72 h after transfection by visualizing GFP expression on an Incucyte Live-Cell Analysis System (Sartorius). The percentage of infection was calculated by dividing the number of GFP-positive cells by the total number of cells. IC_50_ was calculated from infection curves obtained from mock-infected cells by nonlinear regression using “dose–response—inhibition” function in GraphPad Prism 9 Software (Version 9.0.2). SARS-CoV-2 experiments were conducted in Containment Level 3 Laboratory.

### Live virus neutralization (Wuhan)

Neutralization assay was performed using live WT human SARS-CoV-2 strain (SARS-CoV-2-WT) extracted from Trondheim population in Norway and recombinant mCherry-expressing SARS-CoV-2 (SARS-CoV-2-mCherry), provided by the European Virus Archive global (EVAg) and Prof. Andres Merits, respectively (104, 105). To test the ability of albumin-fused ACE2 to block cellular infection, kidney epithelial cells extracted from an African green monkey (Vero E6), cultured in DMEM (Gibco) supplementing heat-inactivated 10% FBS and 1X Penicillin–Streptomycin (Gibco) and maintained at 37 °C in a humidified atmosphere of 5% CO_2_, were seeded at a density of 4 × 10^4^ cells/well in 96-well plates and allowed to adhere overnight. The medium was replaced with DMEM supplemented with 0.2% Bovine Serum Albumin (BSA; Sigma-Aldrich) and 1X Penicillin–Streptomycin (Gibco), containing serial dilutions of WT and QMP albumin-fused ACE2 or QMP albumin-fused CD147 (200–1 μg/mL). The control wells received no fusions. The wells were then infected with SARS-CoV-2-WT or SARS-CoV-2-mCherry at a multiplicity of infection (MOI) of 0.1, or mock-infected. CellTiter-Glo Cell Viability Assay (Promega) was performed 72 h post-infection to determine cell viability. IC_50_ was determined using GraphPad Prism 9 Software (Version 9.0.2), as described.

### Live virus inhibition (BA.5, BQ.1.1, and XBB)

Neutralization assays were performed using live SARS-CoV-2 variants BA.5, BQ.1.1, and XBB isolated at the Norwegian Institute of Public Health (106). To evaluate inhibition of the Omicron variants, ACE2-albumin fusions were diluted, in a 5-fold serial dilution, in virus diluent (MEM containing 2% FCS, 1× Penicillin–Streptomycin, and 25 mM HEPES buffer) in a 96-well deep well plate. In a BSL-3 facility, a viral dose of 100×TCID_50_ was added to each well containing diluted ACE2-albumin fusions, or virus control with only virus diluent. Cell controls in virus diluent were also included. The virus-fusion mixtures were incubated at 37 °C for 1 h, and then added to a 96-well plate containing Vero E6 cells. Vero E6 cells were seeded 1 day prior to the experiment, at a density of 12,000 cells/well in MEM containing 5% FCS and 1× Penicillin–Streptomycin. After 96 h of incubation at 37 °C, the plates were checked for cytopathic effect using a light microscope. The cells were fixed with 80% acetone before transporting out of the BSL3 facility. ELISA detecting the nucleocapsid of SARS-CoV-2 was performed on the fixed cell layer in a BSL2 facility. The ELISA setup consists of a blocking step using PBS with 1% BSA, primary incubation with SARS-CoV-2 nucleocapsid antibody (Sino Biological, 40143-R019), and secondary incubation with Goat anti-rabbit IgG ALP Antibody (Sigma-Aldrich, A3687). Between each incubation step, the plates were washed with wash buffer (PBS with 2% Tween 20). Finally, 1 mg/mL of phosphatase substrate dissolved in diethanolamine buffer was added to the plates, and the absorbance was measured at 405 nm after 40 min. IC_50_ was determined using GraphPad Prism 9 Software (Version 9.0.2), as described.

### Live virus replication inhibition

293T-ACE2-TMPRSS2 cells were seeded into 96-well plates at a density of 1.5 × 10^4^ cells/well 24 h prior to infection. For infections, an isolate of clinical isolate SARS-CoV-2/human/Liverpool/REMRQ0001/2020 (a kind gift from Lance Turtle [University of Liverpool, United Kingdom] and David Matthews and Andrew Davidson [University of Bristol, United Kingdom]) was used. Virus stock was generated in Vero cells expressing human ACE2 and TMPRSS2, as previously described (81). Briefly, DMEM (Gibco) containing 2% FBS and virus at MOI = 0.2 were mixed with serial dilutions of ACE2-albumin fusions and added to the cells for 18 h. Control Regeneron antibodies were used at 2.6 mg/mL. Following infection, the cells were frozen and freeze-thawed prior to cell lysis with 1 volume of buffer containing 0.25% Triton X-100, 50 mM KCl, 100 mM Tris–HCl (pH 7.4), glycerol 40%, and RNAsecure (1/100) for 5 min. Lysates were transferred into a PCR plate for incubation at 95 °C for 5 min to inactivate the live virus. Virus replication was quantified by RT-qPCR, as previously described (107). First, the lysates were diluted (1:50) and then used Luna Universal Probe One-Step kit (NEB) using the following reagents: primer/probes CDC-N1 (IDT) for genomic viral RNA, 18S control standards SARS-CoV-2_N_Positive control RNA (IDT), and 18S standard DNA (a kind gift from Jordan Clarks and James Stewart [University of Liverpool, United Kingdom]). Final concentrations of 500 nM for each primer and 125 nM for the probe were used. RT-qPCRs were run with following program: 55 °C for 10 min and 95 °C for 1 min, followed by 40 cycles of 95 °C denaturation for 10 s and 60 °C extension for 30 s on ABI StepOnePlus PCR System (Life Technologies). RNA copy numbers were obtained from standards and then genomic copies of N normalized to copies of 18S. Data were normalized to 100% of untreated control (negative) and then log transformed. SARS-CoV-2 experiments were conducted in Containment Level 3 Laboratory.

## Data analysis and presentation

GraphPad Prism 9 Software (Version 9.0.2; GraphPad Software, Inc.) was used to analyze data, generate graphs and figures, and perform statistical analysis using unpaired two-tailed *t*-test. Illustrations were created with BioRender.com.

## Supplementary Material

pgad403_Supplementary_Data

## Data Availability

All data and materials in the main text and Supplementary material are available upon reasonable request to the corresponding author for the purposes of reproducing or extending the analysis. All requests will be reviewed to determine whether they are subject to confidentiality and data protection obligations. Data that can be shared will be made available through material transfer agreements.
