# Supplementary material for: A pan-SARS-CoV-2-specific soluble angiotensin-converting enzyme 2-albumin fusion engineered for enhanced plasma half-life and needle-free mucosal delivery
Source: PNAS Nexus. 2023 Nov 28;2(12):pgad403. doi: 10.1093/pnasnexus/pgad403 (PMC10703496; doi:10.1093/pnasnexus/pgad403)
Supplement: pgad403_Supplementary_Data [file pgad403_supplementary_data.pdf]

Supplementary Information for

A pan-SARS-CoV-2-specific soluble angiotensin-converting enzyme 2-albumin fusion engineered for enhanced plasma half-life and needle-free mucosal delivery

Sopisa Benjakul[1,2,3], Aina Karen Anthi[1,2,3†], Anette Kolderup[1,2,3†], Marina Vaysburd[4], Heidrun Elisabeth Lode[1,2,5], Donna Mallery[4], Even Fossum[6], Elisabeth Lea Vikse[6], Anna Albecka[4], Aleksandr Ianevski[7], Denis Kainov[7,8,9], Karine Flem Karlsen[1,2], Siri Aastedatter Sakya[1,2,3], Mari Nyquist-Andersen[1,2,3], Torleif Tollefsrud Gjølberg[1,2,3,5], Morten C. Moe[5], Magnar Bjørås[6], Inger Sandlie[10], Leo C. James[4], and Jan Terje Andersen[1,2,3*]

Affiliations:

[1]Department of Pharmacology, Institute of Clinical Medicine, University of Oslo, Oslo 0372, Norway.

[2]Department of Immunology, Oslo University Hospital Rikshospitalet, Oslo 0372, Norway.

[3]Precision Immunotherapy Alliance (PRIMA), University of Oslo, Oslo 0372, Norway.

[4]Protein and Nucleic Acid Chemistry Division, Medical Research Council, Laboratory of Molecular Biology, Cambridge CB2 0QH, United Kingdom.

[5]Department of Ophthalmology, Oslo University Hospital and University of Oslo, Oslo 0450, Norway.

[6]Department of Virology, Norwegian Institute of Public Health, 0213 Oslo, Norway.

[7]Department of Clinical and Molecular Medicine, Norwegian University of Science and Technology, Trondheim 7491, Norway.

[8]Institute of Technology, University of Tartu, Tartu 50411, Estonia.

[9]Institute for Molecular Medicine Finland, University of Helsinki, Helsinki 00290, Finland.

[10]Department of Biosciences, University of Oslo, Oslo 0371, Norway.

†Contributed equally

*Corresponding author: Jan Terje Andersen.

Email: j.t.andersen@medisin.uio.no

This PDF file includes:

Figures S1 to S6
Tables S1 to S8

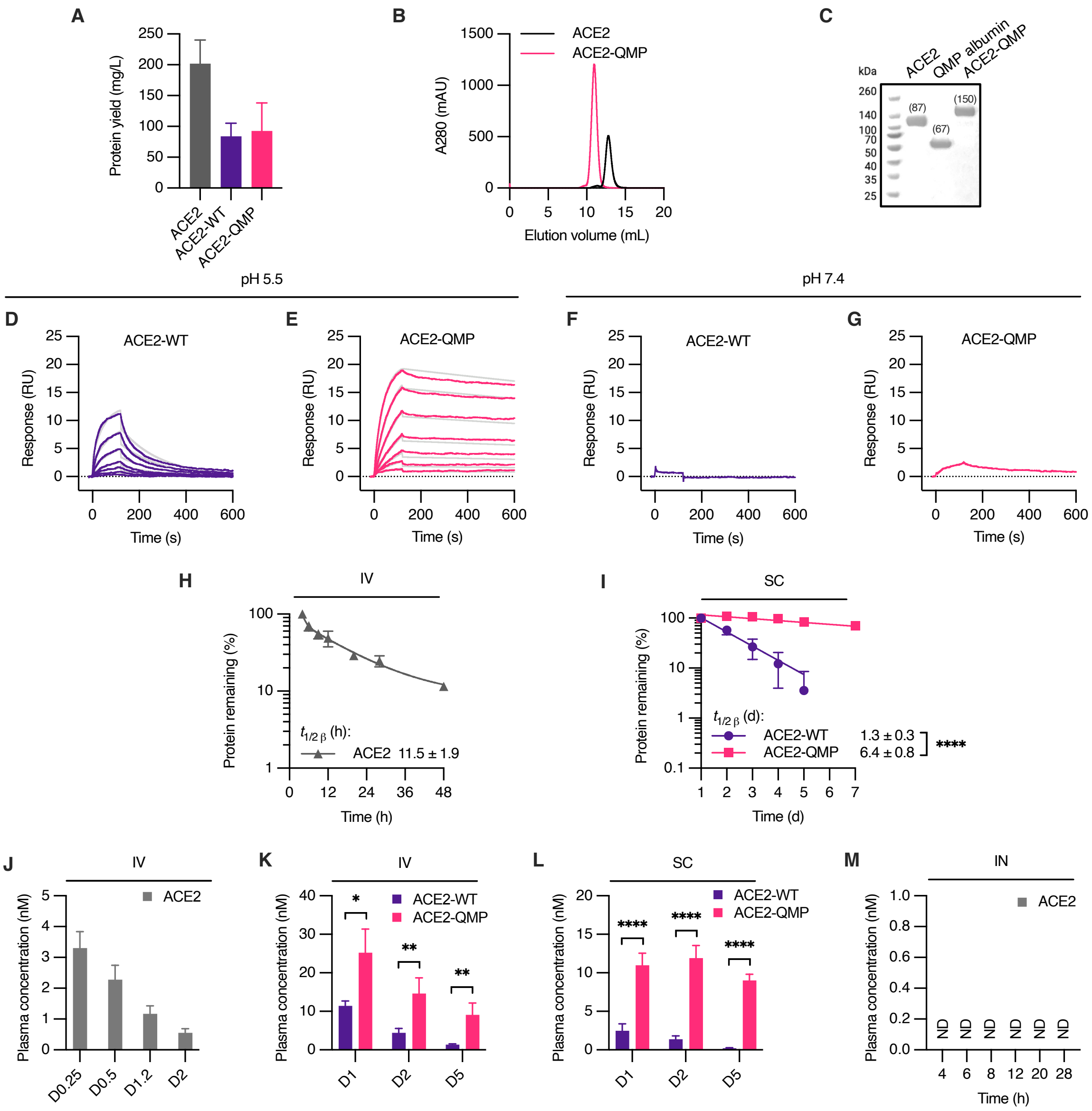

Fig. S1. Design of dimeric ACE2-albumin fusions. (**A**) Yield of purified soluble truncated dimeric human ACE2, ACE2-WT, and ACE2-QMP secreted from Expi293F cells in serum-free suspension upon transient expression (mean ± SD, n = 2–5). (**B**) Representative SEC profile and (**C**) nonreducing SDS-PAGE gel of purified noncovalent dimeric ACE2 or full-length QMP albumin, in comparison to noncovalent dimeric ACE2-QMP. (**D–G**) Representative SPR sensorgrams showing the binding of serial dilutions of the monomeric human FcRn-His injected in over immobilized ACE2-WT and ACE2-QMP at (D and E) pH 5.5 and (F and G) pH 7.4. The fit of the data, from one of the duplicates, to the 1:1 Langmuir binding model is shown. (**H**) Representative biphasic decay curve of unfused ACE2 post-IV administration in homozygous human FcRn Tg32 mice. The data are presented as the percentage of protein remaining in plasma and the average plasma half-life (mean ± SD; n = 5). (**I**) Representative elimination curves of ACE2-WT and ACE2-QMP post-SC administration in hemizygous human FcRn Tg32 mice. The data are presented as the percentage of protein remaining in plasma compared with day 1 and the average plasma half-lives (mean ± SD n = 5). (**J**) Plasma concentrations of unfused ACE2 at 6, 12, 28, and 48 h post-IV administration in homozygous human FcRn Tg32 mice (mean ± SD, n = 5). (**K** and **L**) Plasma concentrations of ACE2-WT and ACE2-QMP on day 1, 2, and 5 (K) post-IV administration in homozygous human FcRn Tg32 mice and (L) post-SC administration in hemizygous human FcRn Tg32 mice (mean ± SD, n = 5). (**M**) Plasma concentrations of ACE2 post-IN administration in homozygous human FcRn Tg32 mice (mean ± SD, n = 5). Unpaired two-tailed t-test was used for statistical analysis, where *P = 0.0137, **P = 0.0095, and ****P < 0.0001.

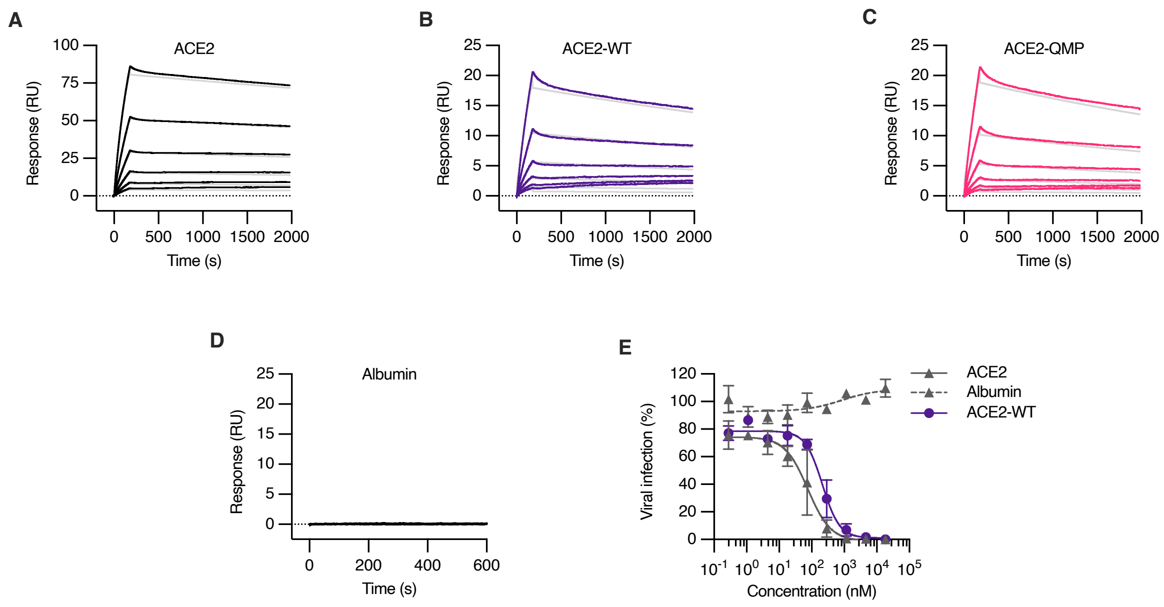

Fig. S2. Viral binding and neutralization of dimeric ACE2-albumin fusions. (**A–D**) Representative SPR sensorgrams showing the binding of serial dilutions of (A) unfused ACE2, (B) ACE2-WT, (C) ACE2-QMP, and (D) WT albumin injected over immobilized monomeric RBD derived from SARS-CoV-2 (Wuhan). The fit of the data, from one of the duplicates, to the 1:1 Langmuir binding model is shown. (**E**) Representative pseudovirus neutralization experiment showing the capacity of ACE2-WT to block cellular infection of Wuhan spike-pseudotyped lentivirus in 293T-ACE2-TMPRSS2 cells, in comparison to unfused ACE2 and WT albumin (mean ± SD, *n* = 2).

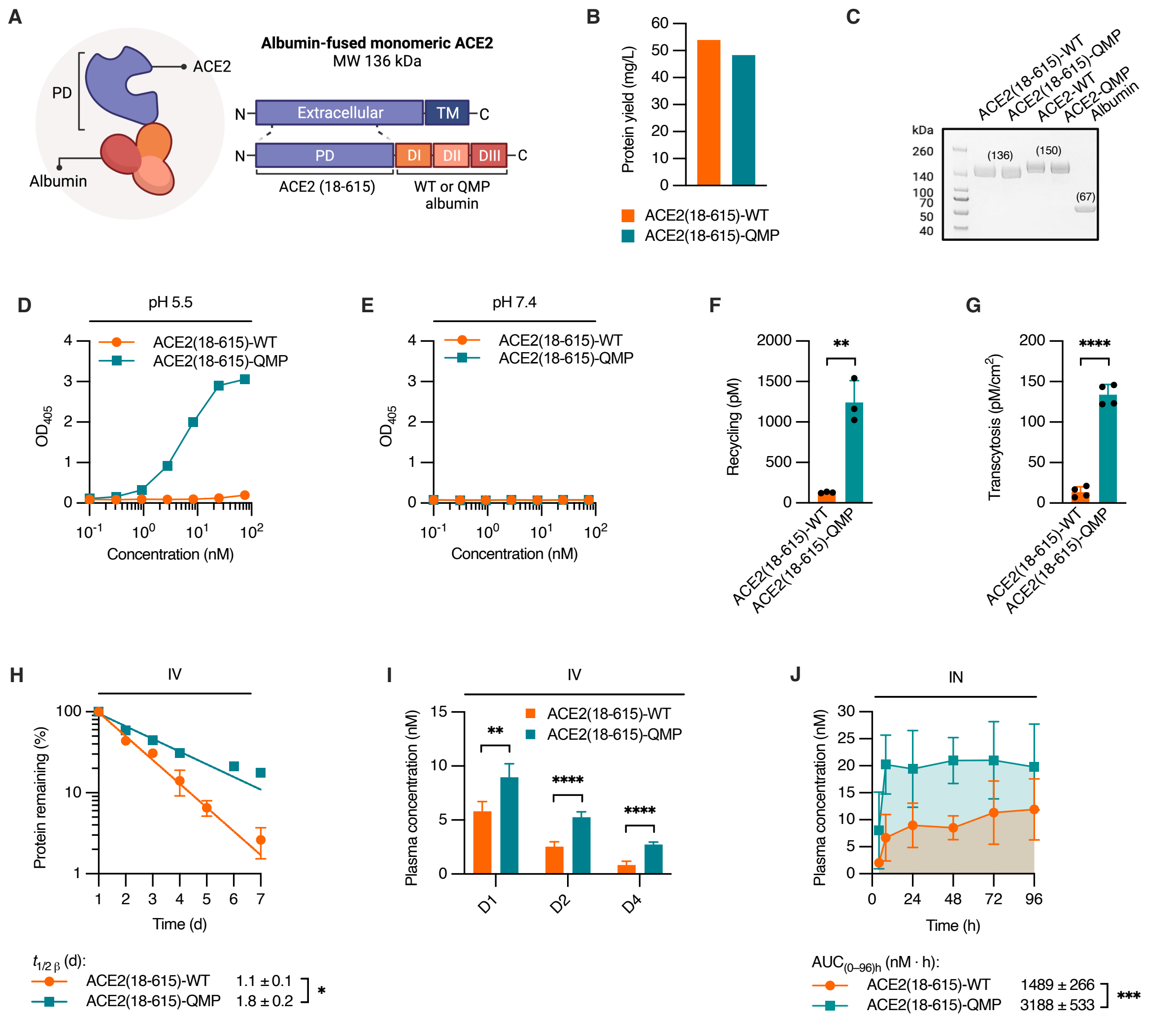

Fig. S3. Design of albumin-fused monomeric ACE2. (**A**) Illustration of the monomeric ACE2-albumin fusions (136 kDa), in which the PD of truncated monomeric ACE2 (residues 18–615) is genetically fused to the N-terminal end of full-length human WT or QMP albumin. The three sub-domains DI, DII, and DIII of albumin are indicated. (**B**) Yield of purified soluble monomeric ACE2(18–615) fused with WT or QMP albumin secreted from Expi293F cells in serum-free suspension upon transient expression (n = 1). (**C**) Nonreducing SDS-PAGE gel of monomeric ACE2-albumin fusions (136 kDa), ACE2(18–615)-WT, and ACE2(18–615)-QMP, in comparison to noncovalent dimeric ACE2-albumin fusions (150 kDa), ACE2-WT and ACE2-QMP, as well as WT albumin (67 kDa). (**D** and **E**) Representative ELISA results showing the pH-dependent binding of ACE2(18–615)-WT and ACE2(18–615)-QMP to human FcRn-His at (D) pH 5.5 and (E) pH 7.4 (mean ± SD, n = 2). (**F**) The recycled amount of monomeric ACE2(18–615)-WT and ACE2(18–615)-QMP in a representative HERA with adherent HMEC-1-FcRn cells (mean ± SD, n = 3). (**G**) The amounts of ACE2(18–615)-WT and ACE2(18–615)-QMP transcytosed from the apical to basolateral side in a representative Transwell assay with polarized MDCKII-FcRn cells (mean ± SD, n = 4). (**H** and **I**) Representative half-life study of monomeric ACE2-albumin fusions in homozygous human FcRn Tg32 mice. (H) Elimination curves of ACE2(18–615)-WT and ACE2(18–615)-QMP post-IV administration. The data are presented as the percentage of protein remaining in plasma compared with day 1 and the average plasma half-lives (mean ± SD; n = 5). (I) Plasma concentrations of ACE2(18–615)-WT and ACE2(18–615)-QMP on day 1, 2, and 4 post-IV administration (mean ± SD, n = 5). (**J**) Representative pulmonary delivery study in homozygous human FcRn Tg32 mice. The data are presented as the plasma concentrations of ACE2(18–615)-WT and ACE2(18–615)-QMP at 4, 8, 48, 72, and 96 h post-IN administration, and the respective AUC (mean ± SD; n = 5). Unpaired two-tailed t-test was used for statistical analysis, where *P = 0.140, **P = 0.0017, ***P = 0.0020, and ****P < 0.0001.

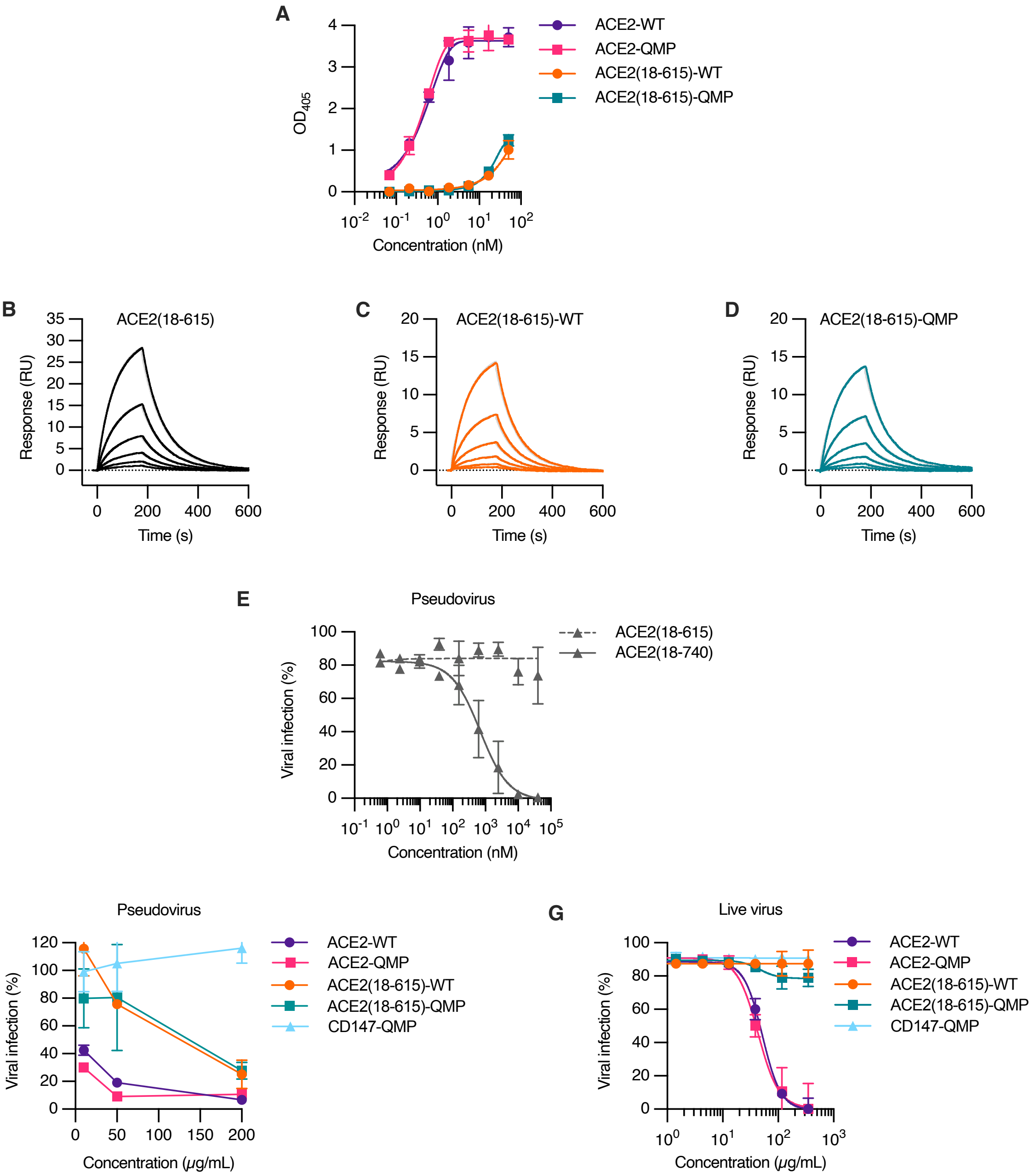

Fig. S4. Effective neutralization of SARS-CoV-2 requires ACE2 dimerization. (A) Representative ELISA showing the binding of dimeric and monomeric ACE2-albumin fusions to derived RBD of Wuhan SARS-CoV-2 (mean ± SD, *n* = 2). (**B–D**) Representative SPR sensorgrams showing the binding of serial dilutions of monomeric (B) unfused ACE2(18–615), (C) ACE2(18–615)-WT, and (D) ACE2(18–615)-QMP injected over immobilized monomeric Wuhan SARS-CoV-2 RBD. The fit of the data, from one of the duplicates, to the 1:1 Langmuir binding model is shown. (**E**) Representative pseudovirus neutralization experiment showing the capacity of unfused monomeric ACE2(18–615) and dimeric ACE2(18–740) to block cellular infection of Wuhan SARS-CoV-2 spike-pseudotyped lentivirus to 293T-ACE2-TMPRSS2 cells (mean ± SD, *n* = 2).(**F** and **G**) Representative neutralization experiments showing the capacity of dimeric and monomeric ACE2-albumin fusions to block cellular infection of Wuhan SARS-CoV-2 (E) spike-pseudotyped lentivirus to 293T-ACE2-TMPRSS2 cells and (F) live virus to Vero E6 cells, in comparison to CD147-QMP (mean ± SD; *n* = 2).

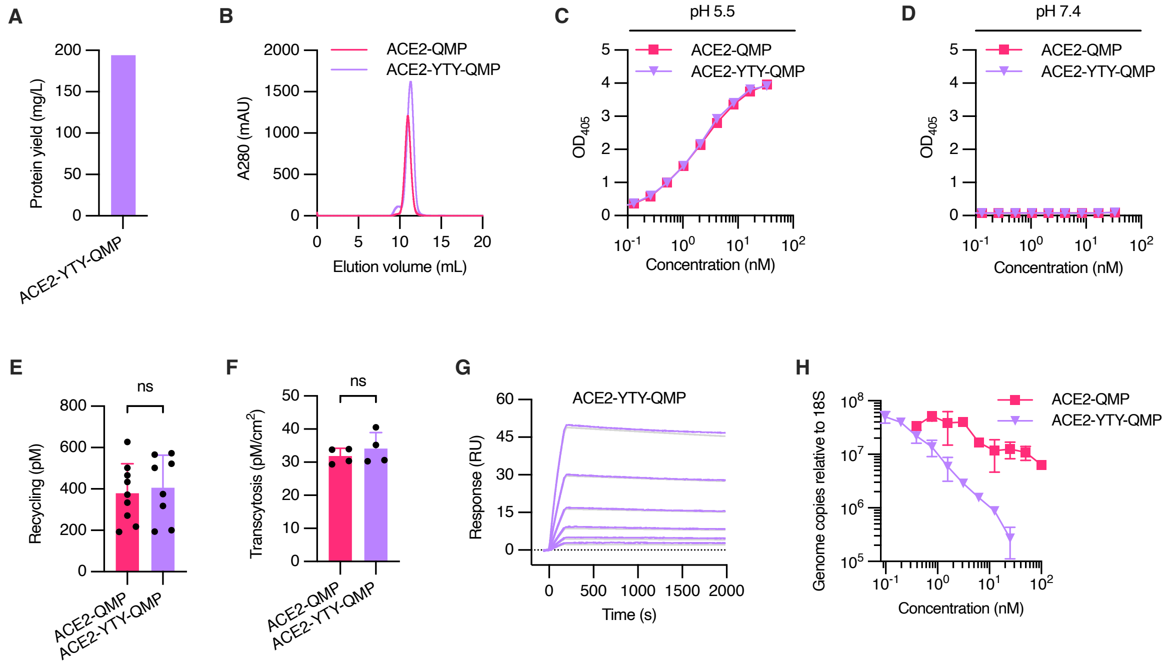

Fig. S5. Design of dimeric ACE2-YTY-QMP with enhanced viral blockage. (**A**) Yield of purified soluble dimeric ACE2-YTY-QMP secreted from Expi293 cells in serum-free suspension upon transient expression (*n* = 1). (**B**) Representative SEC profile of purified ACE2-QMP and ACE2-YTY-QMP. (**C** and **D**) Representative ELISA results showing the pH-dependent binding of ACE2-QMP and ACE2-YTY-QMP to human FcRn-His at (C) pH 5.5 and (D) pH 7.4 (mean ± SD, *n* = 2). (**E**) The recycled amounts of ACE2-QMP and ACE2-YTY-QMP in HERA with adherent HMEC-1-FcRn cells (mean ± SD, *n* = 3; 3 independent experiments). (**F**) The amounts of ACE2-QMP and ACE2-YTY-QMP transcytosed from the apical to basolateral side in a representative Transwell assay with polarized MDCKII-FcRn cells (mean ± SD, *n* = 4). (**G**) Representative SPR sensorgram showing the binding of serial dilutions of ACE2-YTY-QMP injected over immobilized Wuhan SARS-CoV-2 RBD. The fit of the data, from one of the duplicates, to the 1:1 Langmuir binding model is shown. (**H**) Representative neutralization experiment showing the capacity ACE2-QMP and ACE2-YTY-QMP to block cellular infection of live Wuhan SARS-CoV-2 to 293T-ACE2-TMPRSS2 cells (mean ± SD; *n* = 3). Unpaired two-tailed *t*-test was used for statistical analysis, where *ns* = not significant.

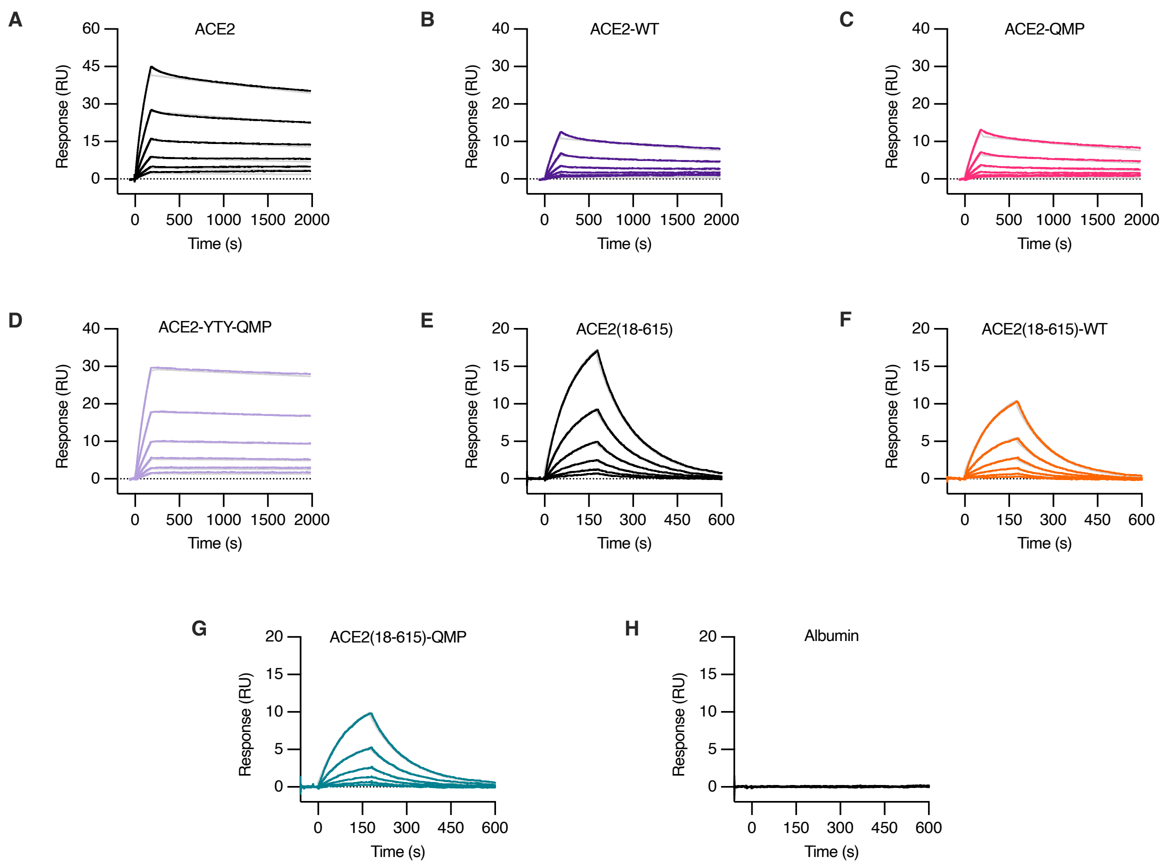

Fig. S6. Binding kinetics of ACE2-albumin fusions to SARS-CoV-2 RBD (B.1.617.2). Representative SPR sensorgrams showing the binding of serial dilutions of (**A**) unfused dimeric ACE2, (**B**) dimeric ACE2-WT, (**C**) dimeric ACE2-QMP, (**D**) dimeric ACE2-YTY-QMP, (**E**) unfused monomeric ACE2(18–615), (**F**) monomeric ACE2(18–615)-WT, (**G**) monomeric ACE2(18–615)-QMP, and (**H**) WT albumin injected over immobilized monomeric SARS-CoV-2 RBD of the B.1.617.2 variant. The fit of the data, from one of the duplicates, to the 1:1 Langmuir binding model is shown.

Table S1. SPR-derived binding kinetics and affinity of dimeric ACE2-albumin fusions to human FcRn[a].

Sample	pH	k_a (10^4 M^{-1} s^{-1})	k_d (10^{-3} s^{-1})	K_D (nM)	X^2 (RU^2)[b]
ACE2-WT	5.5	6.0	6.1	102.4	0.1
	7.4	N/A	N/A	N/A	N/A
ACE2-QMP	5.5	25.4	0.2	0.8	0.1
	7.4	N/A	N/A	N/A	N/A

N/A: Not acquired due to low or no activity.

[a] Kinetic and affinity constants were obtained using a simple first-order (1:1) Langmuir biomolecular interacting model from duplicated runs, where k_a is the association constant, k_d is the dissociation constant, and K_D is the equilibrium dissociation constant.

[b] χ^2 is the chi-square and a measure of the average squared residual (i.e., the difference between the experimental data and the fitted curve).

Table S2. SPR-derived binding kinetics and affinity of monomeric and dimeric unfused ACE2 and ACE2-albumin fusions to SARS-CoV-2 RBD[a].

Sample	Variant	k_a (10^5 M^{-1} s^{-1})	k_d (10^{-3} s^{-1})	K_D (nM)	X^2 (RU^2)[b]
ACE2(18–615)	Wuhan	2.90	24.7	85.1	0.02
	B.1.617.2	2.12	13.4	62.9	0.01
ACE2(18–615)-WT	Wuhan	0.38	12.4	327.0	0.01
	B.1.617.2	0.47	8.3	175.0	0.01
ACE2(18–615)-QMP	Wuhan	2.40	26.0	108.0	0.02
	B.1.617.2	3.21	25.8	80.3	0.01
ACE2	Wuhan	2.61	0.07	0.3	1.61
	B.1.617.2	2.75	0.14	0.4	0.49
ACE2-WT	Wuhan	1.66	0.15	0.9	0.46
	B.1.617.2	1.59	0.21	1.3	0.17
ACE2-QMP	Wuhan	1.05	0.22	2.1	0.23
	B.1.617.2	1.19	0.24	2.0	0.13
ACE2-YTY-QMP	Wuhan	1.89	0.04	0.2	0.32
	B.1.617.2	2.01	0.04	0.2	0.14
Albumin	Wuhan	N/A	N/A	N/A	N/A
	B.1.617.2	N/A	N/A	N/A	N/A

N/A: Not acquired due to low or no activity.

[a] Kinetic and affinity constants were obtained using a simple first-order (1:1) Langmuir biomolecular interacting model from duplicated runs, where k_a is the association constant, k_d is the dissociation constant, and K_D is the equilibrium dissociation constant.

[b] χ^2 is the chi-square and a measure of the average squared residual (i.e., the difference between the experimental data and the fitted curve).

Table S3. Half-maximal inhibition concentrations of monomeric and dimeric unfused ACE2 and ACE2-albumin fusions required for blockage of Wuhan SARS-CoV-2 pseudotyped lentivirus to 293T-ACE2-TMPRSS2 cells and live virus to Vero E6 cells.

Sample	IC_{50} (nM)	
	Pseudovirus neutralization	Live virus neutralization
ACE2(18–615)	N/A	-
ACE2(18–615)-WT	725.3[a]	N/A
ACE2(18–615)-QMP	692.7[a]	N/A
ACE2	7.9 ± 0.4[b]	-
ACE2-WT	20.4 ± 1.3[b]	293.0[a]
ACE2-QMP	15.0 ± 2.8[b]	255.0[a]
Albumin	N/A	-
CD147-QMP	N/A	N/A

N/A: Not acquired due to low or no activity.

[a] The data represent mean from a representative experiment (n = 3).

[b] The data represent mean ± SD from two independent experiments (n = 3).

Table S4. Half-maximal inhibition concentrations of dimeric ACE-YTY-QMP required for blockage of SARS-CoV-2 pseudotyped lentivirus to 293T-ACE2-TMPRSS2 cells.

Variant	Sample	IC_{50} (nM)
Wuhan	ACE2-WT	15.65
	ACE2-QMP	9.24
	ACE2-YTY-QMP	0.78
B.1.617.2	ACE2-WT	6.69
	ACE2-QMP	3.42
	ACE2-YTY-QMP	0.90
B.1.1.529	ACE2-WT	1.05
	ACE2-QMP	0.88
	ACE2-YTY-QMP	0.10

Table S5. Half-maximal inhibition concentrations of dimeric ACE-YTY-QMP required for blockage of live Wuhan SARS-CoV-2 to 293T-ACE2-TMPRSS2 cells.

Sample	IC_{50} (nM)
ACE2-WT	N/A
ACE2-QMP	N/A
ACE2-YTY-QMP	58.6

N/A: Not acquired due to low activity.

Table S6. Half-maximal inhibition concentrations of dimeric ACE-YTY-QMP required for blockage of live SARS-CoV-2 variants (BA.5, BQ.1.1, and XBB) to Vero E6 cells.

Variant	Sample	IC_{50} (nM)
BA.5	ACE2	3.67
	ACE2-QMP	5.77
	ACE2-YTY-QMP	0.89
BQ.1.1	ACE2	11.23
	ACE2-QMP	23.21
	ACE2-YTY-QMP	3.22
XBB	ACE2	26.40
	ACE2-QMP	33.16
	ACE2-YTY-QMP	4.37

Table S7. Overview of the designed vectors of monomeric and dimeric ACE2-albumin fusions.

Fusion	GenBank accession no.	Amino acid
ACE2(18–615)-WT	ACE2: NP_068576.1<br>Albumin: CAA23754.1	ACE2: 18–615 (QSTI-PYAD)<br>Albumin: 25–609 (DAHK-ALGL)
ACE2(18–740)-WT	ACE2: NP_068576.1<br>Albumin: CAA23754.1	ACE2: 18–740 (QSTI-PPVS)<br>Albumin: 25–609 (DAHK-ALGL)
ACE2(18–615)-QMP	ACE2: NP_068576.1<br>Albumin: CAA23754.1	ACE2: 18–615 (QSTI-PYAD)<br>Albumin: 25–609 (DAHK-ALGL)
ACE2(18–740)-QMP	ACE2: NP_068576.1<br>Albumin: CAA23754.1	ACE2: 18–740 (QSTI-PPVS)<br>Albumin: 25–609 (DAHK-ALGL)

Table S8. Overview of mutations in the spike and derived RBD of SARS-CoV-2 variants.

Variant	Mutations in spike	Mutations in RBD
B.1.1.7<br>(Alpha)	ΔH69-V70<br>ΔY144<br>N501Y<br>A570D<br>P681H<br>T716I<br>S982A<br>D1118H	N501Y
B.1.351<br>(Beta)	L18F<br>D80A<br>D215G<br>L242H<br>R246I<br>K417N<br>E484K<br>N501Y<br>D614G<br>A701V	K417N<br>E484K<br>N501Y
P.1<br>(Gamma)	L18F<br>T20N<br>P26S<br>D138Y<br>R190S<br>K417T<br>E484K<br>N501Y<br>H655Y<br>T1027I	K417T<br>E484K<br>N501Y
B.1.617.2<br>(Delta)	T19R<br>G142D<br>ΔE156-F157<br>R158G<br>L452R<br>T478K<br>D614G<br>P681R<br>D950N	L452R<br>T478K
B.1.1.529<br>(Omicron)	A67V<br>ΔH69-V70<br>T95I<br>G142D<br>ΔV143-Y144-Y145<br>ΔI211<br>L212I<br>ins214EPE<br>G339D<br>S371L<br>S373P<br>S375F<br>K417N<br>N440K<br>G446S<br>S477N<br>T478K<br>E484A<br>Q493K<br>G496S<br>Q498R	

	N501Y<br>Y505H<br>T547K<br>D614G<br>H655Y<br>N679K<br>P681H<br>N764K<br>D796Y<br>N856K<br>Q954H<br>N969K	
